# Genetic and epigenetic regulation of growth, reproduction, disease resistance and stress responses in aquaculture

**DOI:** 10.3389/fgene.2022.994471

**Published:** 2022-11-02

**Authors:** Zhanjiang Liu, Tao Zhou, Dongya Gao

**Affiliations:** ^1^ Department of Biology, College of Arts and Sciences, Syracuse University, Syracuse, NY, United States; ^2^ Fujian Key Laboratory of Genetics and Breeding of Marine Organisms, College of Ocean and Earth Sciences, Xiamen University, Xiamen, China

**Keywords:** DNA methylation, epigenetic regulation, genome, QTL, fish, shellfish, aquaculture

## Abstract

Major progress has been made with genomic and genetic studies in aquaculture in the last decade. However, research on epigenetic regulation of aquaculture traits is still at an early stage. It is apparent that most, if not all, aquaculture traits are regulated at both genetic and epigenetic levels. This paper reviews recent progress in understanding of genetic and epigenetic regulation of important aquaculture traits such as growth, reproduction, disease resistance, and stress responses. Although it is challenging to make generalized statements, DNA methylation is mostly correlated with down-regulation of gene expression, especially when at promoters and enhancers. As such, methylation of growth factors and their receptors is negatively correlated with growth; hypomethylation of genes important for stress tolerance is correlated with increased stress tolerance; hypomethylation of genes important for male or female sex differentiation leads to sex differentiation into males or females, respectively. It is apparent that environmental regulation of aquaculture traits is mediated at the level of epigenetic regulation, and such environment-induced epigenetic changes appeared to be intergenerationally inherited, but evidences for transgenerational inheritance are still limited.

## 1 Introduction

Important performance and production traits for aquaculture include growth rate, feed conversion efficiency, disease resistance, stress tolerance such as low oxygen tolerance, reproductive success, harvestability, and processing yields, among many others (Gjedrem, 2012; Boyd, 2015; Abdelrahman et al., 2017; Zhong et al., 2017; Li et al., 2019; Houston et al., 2020; Xu et al., 2022). Of these traits, disease resistance continues to be the top priority for aquaculture production because disease problems constitute the largest single cause of economic losses in aquaculture (Mzula et al., 2021; Naylor et al., 2021). Most, if not all, of these traits are regulated at both genetic and epigenetic levels, and as such, understanding of genetic and epigenetic regulation of aquaculture traits is of high priority for aquaculture genomics and genetics research (Abdelrahman et al., 2017; Granada et al., 2018; Rexroad et al., 2019; Roy et al., 2021; Ren et al., 2022).

The central dogma has guided biological research for over half of a century. However, research advances in the last 20 years have provided information for the more complete interpretation of the central dogma. These included the huge regulatory functions of non-coding and small RNAs, epigenetic regulation through DNA methylation, and epigenetic regulation through histone modifications. This review will focus on DNA methylation and its regulation of genomic expression related to aquaculture traits. While research is very active with non-coding RNAs and histone modifications with aquaculture species, systematic knowledge of how such mechanisms are involved in the control of aquaculture traits is yet to be published, and as such, we will only briefly discuss epigenetic regulation through histone modifications.

**FIGURE 1:**
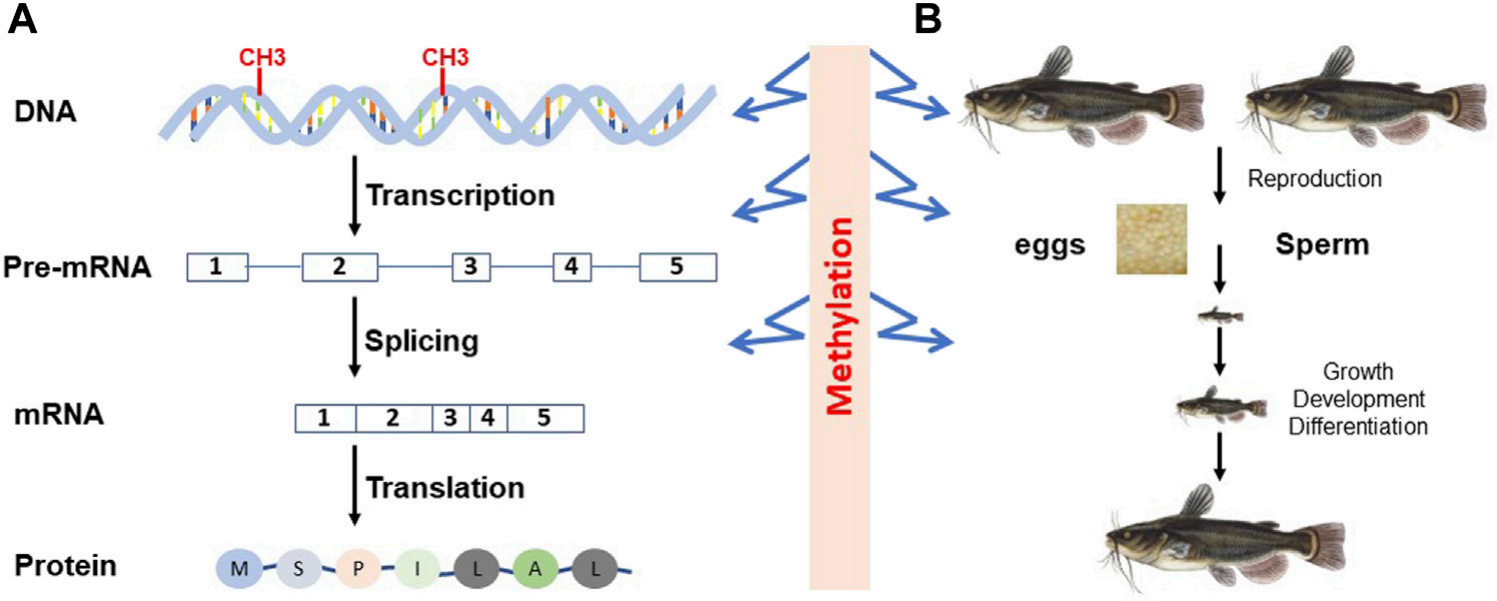
Epigenetic regulation of gene expression in the context of central dogma **(A)** and organismal development **(B)**. At the molecular level, DNA methylation can affect transcription and mRNA splicing, leading to different types or different amounts of transcripts. At the organismal level, DNA methylation is involved in gamete production, embryonic and later development, and differentiation.

In eukaryotic organisms, cytosine methylation is the primary form of DNA methylation, and 5 mC is the major methylated form of cytosine. As shown in Figure 1, DNA methylation can insert its roles in the regulation of genome expression at the level of transcription through activation or repression of transcription, thereby having an impact on the types of transcripts and their amounts, or at the level of posttranscription. At the organismal level, DNA methylation can affect development, growth, differentiation, reproduction, and most other processes throughout the life cycle of organisms (for reviews, see Moore et al., 2013; Zeng and Chen, 2019).

Although DNA methylation is stably maintained in somatic tissues, its patterns and levels show dynamic changes during development. In mammalian systems, the genome undergoes two waves of global demethylation and remethylation: One in the germline, initiated with the erasure of global methylation in primordial germ cells and completed with the establishment of sex-specific methylation patterns during later stages of germ cell development; and the second after fertilization, including the erasure of most methylation marks inherited from the gametes and the subsequent establishment of the embryonic methylation pattern (Cavalli and Heard, 2019; Zeng and Chen, 2019). Histone marks and 3D genome organization are also reprogrammed in the germline and after fertilization (Eckersley-Maslin et al., 2018). In zebrafish, the primordial germ cells (PGCs) undergo significant DNA methylation reprogramming during germ cell development (Jessop et al., 2018; Marchione et al., 2021); the methylome of PGCs is reset to an oocyte/ovary-like pattern at 9 days post fertilization (9 dpf), suggesting that methylation reprogramming is required for zebrafish sex transition (Wang et al., 2021). It was demonstrated that zebrafish achieve a totipotent chromatin state at zygotic genome activation (ZGA) through paternal genome competency and maternal genome DNA methylation reprogramming (Potok et al., 2013). Similar developmental regulation processes for epigenetic factors have been observed in a non-model teleost (Wang et al., 2022; Yang et al., 2022).

In the last decade, drastic progress has been made in aquaculture genomics and genetics research. Major milestones included the production of whole genome sequences for many aquaculture species. Many of these genome sequences are of high quality. The availability of high-quality genome sequence assemblies allowed mapping of QTLs controlling important performance and production traits to chromosomal locations, with information of tightly linked markers, especially sequence-tagged single nucleotide polymorphic (SNP) markers. Such high-quality genome sequence assemblies also allowed mapping of methylated bases to genome locations in relation to performance traits. At the same time, high throughput RNA-Seq has allowed qualitative as well as quantitative assessment of genome-wide transcription in relation to genomic sequence variations and epigenomic regulation. This review will summarize recent studies of aquaculture traits at genetic and epigenetic levels, with a focus of DNA methylation.

## 2 DNA methylation and epigenetic regulation

DNA methylation is catalyzed by DNA methyltransferases, and its regulation of gene expression is materialized by the differential binding affinities of methylated DNA to various transcriptional factors and methylated DNA binding proteins, as compared to those of unmethylated DNA. Most often, methylation at the transcriptional regulatory sequences such as the promoters has a negative impact to the transcription of the involved gene(s). Therefore, significantly lower levels of CpG methylation have been observed around transcriptional start sites (TSS). This section reviews general information of DNA methylation and its regulation at various levels.

### 2.1 DNA methyltransferases and methylated DNA binding proteins

DNA methyltransferases (DNMTs) regulate the transfer of methyl groups from S-adenosylmethionine to cytosine residues on genomic DNA. In mammals there are four members of the DNMT family, including DNMT1, DNMT3A, DNMT3B, and DNMT3L, of which DNMT3L does not harbor any transferase activities (Jin and Robertson, 2013), but is essential for establishment of maternal genomic imprints in the growing oocyte and at dispersed repeated sequences. In addition, DNMT2 exists in most, if not all, eukaryotic organisms as a homologue of DNA methyltransferase. It has all the sequence characteristics of a cytosine methyltransferase but has not been demonstrated to carry any methyltransferase activities. DNMT1 has greater methyltransferase activity on hemimethylated substrates and therefore has been assigned into the category of maintenance methyltransferase although it also methylates unmethylated DNA. DNMT3A and DNMT3B are regarded as *de novo* methyltransferases because they do not require hemi-methylated DNA to function and transfer methyl groups to mainly non-methylated cytosine residues (for a review, see Goll and Bestor 2005). In zebrafish, there are eight DNMT enzyme orthologues to the mammalian counterparts (Takayama et al., 2014; Balasubramanian et al., 2019); its DNMT1 is related to mammalian DNMT1, maintaining the methylated DNA created during replication; its DNMT2 is homologous to the mammalian DNMT2; It has a unique function as tRNA transferases catalyzing the methylation at 38th position in tRNA^AspGUC^. Zebrafish DNMT6 and DNMT8 are related to mammalian DNMT3a; its DNMT4 is related to mammalian DNMT3b; and DNMT3, DNMT5, and DNMT7 are unique in fishes (Balasubramanian et al., 2019). DNA methyltransferases have also been studied in other fishes such as Japanese rice fish (Dasmahapatra and Khan, 2015), mandarin fish (Zhou et al., 2021), fathead minnow (Wood et al., 2016), and goldfish (Zhang et al., 2014).

Several methylated DNA binding proteins have been identified, whose expression is involved in the epigenetic regulation through DNA methylation. These included methyl-CpG binding protein 2 (MeCP2), and methyl-CpG-binding domain proteins 1 (MBD1) and 2 (MBD2). MeCP2 is believed to be a global repressor of methylated promoters (Cross et al., 1997), and MDB1 and MDB2 have also been reported to bind with higher affinity to methylated CpG sites than to unmethylated sites (Goll and Bestor, 2005). To date, methylated DNA binding proteins have been studied with aquatic animals only in model species such as zebrafish (Gao et al., 2015; Nozawa et al., 2017).

### 2.2 DNA demethylation

The reverse of DNA methylation is DNA demethylation that can happen passively or actively. Functional deficiency in maintenance of methylation can lead to replication-dependent dilution of 5 mC, which is known as passive DNA methylation (for a review, see Zeng and Chen, 2019). Active DNA demethylation involves rapid loss of DNA methylation by removal of the methyl group from 5 mC. The first step of active demethylation involves oxidation of 5 mC by the ten-eleven translocation (TET) family of enzymes, TET1, TET2, and TET3, which oxidizes 5 mC into 5-hydroxymethylcytosine (5hmC), 5-formylcytosine (5 fC), and 5-carboxylcytosine (5caC) (Tahiliani et al., 2009; Ito et al., 2010, 2011; He et al., 2011). These oxidized products serve as intermediates of DNA demethylation. They are repaired into unmodified C, achieving demethylation (reviewed by Zeng and Chen, 2019).

### 2.3 Major methylation sites in the context of genomic DNA

**FIGURE 2:**
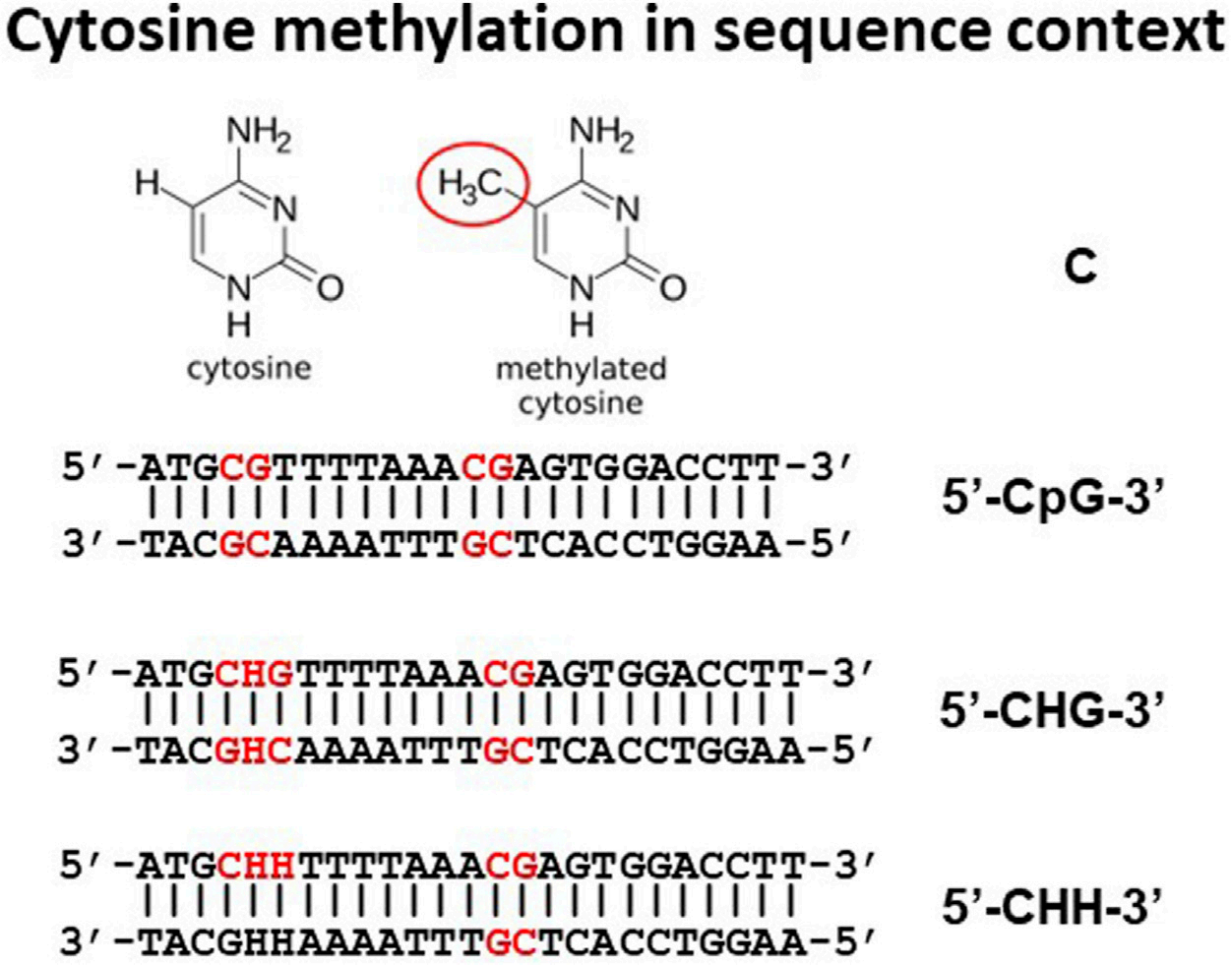
Cytosines within DNA can be within CpG, CHG, or CHH context where H is A, C, or T.

Although methylation of adenine can also happen, we will focus this review on methylation of cytosine. Depending on the sequence context where base C is situated, the chances of being methylated vary greatly (Figure 2). The position of C in a sequence can be in three different situations: 1) CG motifs; 2) CHG motifs where H is A, C, or T; and 3) CHH, where H is A, C, or T. In many fish species studied to date, approximately 70%–80% CpG sites were found to be methylated. For instance, 74.5%–78.4% of all C’s at the CpG sites were methylated in channel catfish (Yang et al., 2022). This is similar to those observed in other vertebrate animal species, slightly higher than that seen in mice (74%) (Feng et al., 2010) and tilapia (69.60%) (Wan et al., 2016), but slightly lower than that in zebrafish (80.3%) (Feng et al., 2010). Methylation rate at CHG and CHH sites are much less frequent, at only miniscule scale as compared to methylation at CpG sites. For example, only 0.3%–0.4% of Cs within the CHG and CHH context were methylated in channel catfish (Yang et al., 2022). Similarly, only 1.22% and 0.91% of Cs within CHG and CHH context were methylated in zebrafish (Feng et al., 2010), 0.47% and 0.57% of Cs within CHG and CHH were methylated in tilapia (Wan et al., 2016). However, methylation rates at CHG and CHH sites is much higher in plants (Kenchanmane Raju et al., 2019).

In contrast to the situations with teleost fish, methylation in invertebrate aquaculture species such as mollusks and crustaceans appeared to be quite different in two aspects: 1) the levels of methylation in crustaceans and bivalves appear to be much lower than those in teleost fish; and 2) DNA methylation in these invertebrate species appears to be predominantly found in gene bodies (Gavery and Roberts, 2013; Gavery and Roberts, 2014). For example, about 15% of CG (1.8% total cytosines) in the Pacific oyster *Crassostrea gigas* genome are methylated (Gavery and Roberts, 2013), which is similar to those in gastropod (snails have ∼2% of cytosines being methylated) (Fneich et al., 2013), but much lower than those observed with vertebrate animals. With marbled crayfsh, *Procambarus virginalis*, the global 5-methylcytosine level was 2.78% at mid-embryonic development and decreased slightly to 2.41% in 2-year-old adults (Vogt, 2022). Methylation levels are even lower with micro-crustaceans. Average percentage CpG methylation was below 1% (Kvist et al., 2018) or just slightly above 1% with *Daphnia magna* (Hearn et al., 2021). Using whole genome bisulfite sequencing, Kvist et al. (2018) demonstrated that DNA methylation in *Daphnia* is mainly enriched within the coding regions of genes, with the highest methylation levels observed at exons 2–4, in contrast to the situations of vertebrates whose genomes are globally methylated. Significant negative correlation between gene family size and the degree of methylation was observed with *Daphnia*, suggesting that gene body methylation may help regulate gene family expansion and functional diversification of gene families leading to phenotypic variations (Asselman et al., 2016).

### 2.4 Regulation of gene expression through DNA methylation

Epigenetics refers to heritable changes in gene expression and phenotype that arise “on top of” or “in addition to” primary DNA sequence. After almost half a century since the term was published, epigenetics has now become one of the hottest research areas in biology because it is involved in essentially all biological processes and often serves as a mechanism of control and regulation. These processes span from life to death involving embryonic development, cell cycle control, growth and development, differentiation, responses to diseases and other environmental factors (Venney et al., 2021), and regulation of aging and cancer (for review, see Skvortsova et al., 2018; Zeng and Chen, 2019; Vidaurre and Chen, 2021; Fu et al., 2020).

A well-established concept of epigenetic regulation is that DNA methylation regulates gene expression by inhibiting the binding of transcription factor(s) to DNA or recruiting proteins involved in gene repression, thereby repressing transcription (Jones, 2012; Moore et al., 2013). Because this is well established and almost over-used concept, we will not further elaborate on this. However, regulation of gene expression by DNA methylation is more complex than this simple notion. In fact, DNA methylation can not only repress transcription, but also activate transcription. For example, transcriptional anti-silencing factors SUVH1 and SUVH3 bind to methylated DNA and recruit the DNAJ proteins to enhance proximal gene expression, thereby encountering the repressive effects of transposon insertion near genes (Harris et al., 2018).

How DNA methylation regulates transcription is complex depending on where and when DNA methylation occurs in relation to gene structure and development. The promoter-region hypermethylation events are especially critical and can frequently serve as alternative mechanisms for coding-region mutations for loss of key gene function (Herman and Baylin, 2000). CpG methylation in promoters are negatively correlated with transcription. As such, most CpG islands when located at transcription start sites are not methylated (Jones 2012). Hypermethylation of the promoter and coding region can inhibit gene expression, while demethylation of the promoter and coding region can activate gene expression (Han et al., 2021a). However, intragenic methylation of CpG sites can activate transcription (Jones, 2012), but such positive correlation of intragenic methylation and transcriptional activation may be tissue specific. Intragenic methylation at CH sites is positively correlated with gene expression in human embryonic cells but is negatively correlated with gene expression in the brain (Luo et al., 2018).

### 2.5 Regulation of recombination through DNA methylation

Genes are not distributed evenly in the genome. Instead, genomes are divided into gene-rich euchromatin regions and repeat-rich heterochromatin regions. Typically, chromosome regions such as centromeres, telomeres are composed almost purely of repetitive sequences. In addition, ribosomal (rRNA) and transfer RNA (tRNA) loci are composed of tandem repeats of these structural RNA genes. Transposable elements can be highly repetitive but in teleost fish, the most abundant types of transposable elements are Tc1/mariner type of transposons that are dispersed throughout the genomes. Repeat-containing regions undergo little or no meiotic crossover recombination.

DNA methylation is a widespread epigenetic mark of repeat sequences associated with heterochromatin in eukaryote genomes. DNA methylation repress meiotic recombination. Recombination rates in heterochromatin regions are generally very low. For instance, genomic regions surrounding the centromeres are generally enriched in DNA methylation and histone modifications such as H3K9me2 (Simon et al., 2015), and there is no recombination in centromeric regions (Termolino et al., 2016). However, the causal relationship of DNA methylation and recombination is not fully demonstrated. The repressive roles of methylation on meiotic recombination in euchromatic regions have been demonstrated, but additional factors may be involved in controlling the suppression of recombination in heterochromatin. For instance, in *Arabidopsis*, deficiency in DNA methylation increased meiotic crossover rates in euchromatic but not in heterochromatic regions (Melamed-Bessudo and Levy, 2012).

### 2.6 Regulation of alternative splicing through DNA methylation

Alternative splicing is an evolutionarily conserved mechanism that increases transcriptome diversity by producing multiple mRNA products from a single gene. In humans, >90% of genes have alternatively spliced transcripts (Wang et al., 2008). In fish species, the proportion of genes with alternative splicing is probably lower. In channel catfish, approximately 39% of genes were found with alternative splice transcripts after infection with *Edwardsiella ictaluri* (Tan et al., 2018a). Methylation levels in exons are higher than in introns, and alternative exons display lower levels of DNA methylation than constitutively spliced exons, suggesting methylation is involved in alternative splicing (Lev Maor et al., 2015). Such regulation of alternative splicing is believed to be mediated by CCCTC-binding factor (CTCF). Intragenic CTCF binding sites, particularly those proximal to splice junctions, influence pre-mRNA splicing decisions, and thus mediate alternative exon or intron inclusion (for a review, see Alharbi et al., 2021). DNA methylation at CpG sites within CTCF target sites can prevent CTCF binding, thereby regulating alternative splicing. Such regulatory processes also involve MeCP2 and TET1. DNA methylation mediates opposing effects on the role of CTCF and MeCP2 binding to DNA and subsequently regulation of pre-mRNA splicing, although the detailed mechanisms are not fully understood (Alharbi et al., 2021).

## 3 Review of recent progress of genetic and epigenetic studies of aquaculture traits

Most of the studies of epigenetic regulation were conducted in humans, rodents, and model organisms. Epigenetic research with aquaculture species, compared to those in model species, are relatively recent and limited, but more and more researchers are turning their attention to this hot research area (Metzger and Schulte, 2016; Gavery and Roberts, 2017). Understanding of epigenetic regulation of various traits will lead to practical applications in aquaculture with various approaches such as epigenomic editing (for a recent review, see Nakamura et al., 2021), environmental manipulation, and epigenetic selection (Metzger and Schulte, 2016).

### 3.1 Reference genome sequences as resources for genetic and epigenetic studies

According to FAO (https://www.fao.org/documents/card/en/c/ca9229en/), aquaculture produced an annual total of over 82 million metric tons of seafood, with finfish contributing over 54 million tons, crustacean contributing over 9.3 million tons, and molluscs contributing over 17.5 million tons. The major finfish, crustaceans and molluscs species of the world aquaculture are summarized in Table 1. Genome sequences for many of the major aquaculture species have been produced. However, well-assembled genome sequences with annotations are still limited. Currently, there are almost 900 eukaryotic genomes that have been annotated at the National Center for Biotechnology Information (NCBI), and it is increasing at a speed of about 150 additional species per year (https://www.ncbi.nlm.nih.gov/genome/annotation_euk/). The annotated genomes to date included 142 fish species, 42 “other vertebrates”, and 69 “other invertebrates” where many of the aquaculture species belong. Despite these impressive numbers, genome sequences for many of the major aquaculture species are yet to be annotated, as summarized in Table 1. Genome annotation is important because well annotated reference genome sequences facilitate genetic and epigenetic research allowing aquaculture traits to be studied more effectively and efficiently.

**TABLE 1:** Major aquaculture species of finfish, crustacean, and molluscs in the world and their whole genome sequence resources.

Species groups	Species involved	% Of world production	References of annotated genomes
Finfish			
Carps (Cypriniformes)	Grass carp, silver carp, **common carp**, catla, bighead carp, **goldfish**, rohu carp, **Wuchang bream**, black carp, and other cyprinids	51.1%	Xu et al. (2014); Chen et al. (2019); Liu H. et al. (2021)
Tilapias	**Nile tilapia**, **blue tilapia**, and other tilapia species	10.2%	Tao et al. (2021); Conte et al. (2017)
Catfishes	**Striped catfish**, Clarias spp., **yellow catfish**, **channel catfish**	7.5%	Liu P. et al. (2016)
			Gong et al. (2018)
			Gao et al. (2021)
Salmonids	**Atlantic salmon, rainbow trout, pink salmon, chum salmon, coho salmon, sockeye salmon, Chinook salmon**	6.1%	Lien et al. (2016); Berthelot et al. (2014)
Milkfish	**Milkfish**	2.4%	
All other finfishes	Flatfishes such as Atlantic halibut, Pacific halibut, **Japanese flounder, half-tongue smooth; Atlantic cod**	16.4%	Chen et al. (2014); Shao et al. (2017); Star et al. (2011)
Crustaceans			
Shrimps	***Penaeus vannamei, P. monodon, P. japonicus, P. chinensis***	60.9	Zhang et al. (2019), Uengwetwanit et al. (2021); Wang et al. (2022)
Red swamp crayfish	***Procambarus clarkii***	18.2	Xu et al. (2021)
Chinese mitten crab	*Eriocheir sinensis*	8.1	
Oriental river prawn	*Macrobrachium nipponense and Macrobrachium rosenbergii*	5.0	Jin et al. (2021)
Molluscs			
Oysters	***Crassostrea gigas***, ***C. virginica***	33.2	Zhang et al. (2012); Gómez-Chiarri et al. (2015)
Japanese carpet shell	*Ruditapes philippinarum*	23.6	
Scallops	***Mizuhopecten yessoensis*** *and various species in Pectinidae*	11.0	Wang et al. (2017a)
Sea mussels	Various species in Mytilidae	6.9	Jin et al. (2017)
Constricted tagelus	*Sinonovacula constricta*	4.9	Ran et al. (2019)
Blood cockle	*Anadara granosa*	2.5	
Chilean mussel	*Mytilus chilensis*	2.1	

Source: Food and Agricultural Organization of the United Nations (https://www.fao.org/documents/card/en/c/ca9229en/).

Bold species are those whose genome sequences have been annotated at NCBI.

### 3.2 Genetic and epigenetic regulation of growth

Growth is among the most important traits for aquaculture production. However, because growth can be selected readily by phenotypes, genetic and genomic work on growth QTL was limited. Nonetheless, genomic work allowed identification of novel growth controlling genes (for review, see Abdelrahman et al., 2017). For example, significant growth QTL have been identified from tilapia (Liu et al., 2014), channel catfish (Li et al., 2018), and Asian seabass (Wang et al., 2019). Interestingly, in eastern oysters, Zeng and Guo (2022) identified a set of genes that are both important for biomineralization and growth. With triploid sea cucumber *Apostichopus japonicus*, the term ribosome production was enriched in fast growing sea cucumbers, and a set of 11 significant differential metabolites were found to be associated with growth advantage (Xie et al., 2022).

A few studies of epigenetic regulation of growth trait have been conducted with fish and shellfish species. Methylation differences were observed between slow and fast muscle in *Takifugu rubripes* (Wang et al., 2021). In allotriploid carps, heterosis and growth are regulated by DNA methylation (Ren et al., 2022). However, the detailed relationship between DNA methylation and gene expression can be complicated. With large yellow croaker, dynamic alterations of methylation of growth-related genes and their expression were observed after starvation treatment, but the correlation of methylation and expression was not consistently observed (Zhang et al., 2019). Increased growth in the interspecific hybrid of snakehead fish was found to be correlated with reduced methylation in the hybrid fry (Ou et al., 2019). In tilapia, increased methylation was observed at the promoter of growth hormone (GH) gene in females, but not in males, correlated with expression of the GH gene and growth performance (Zhong et al., 2014), suggesting epigenetic regulation of growth differences between males and females (Podgorniak et al., 2019). In triploid sea cucumber (*Apostichopus japonicus*), 23 genes (such as Guf1, SGT, Col5a1, HAL, HPS1, etc.) exhibited correlation between levels of promoter methylation and levels of expression, suggesting functional interactions of promoter methylation and growth in triploid sea cucumbers (Han et al., 2021a). DNA methylation is believed to regulate gene expression in polyploid organisms (for a review, see Osborn et al., 2003), and such regulation by DNA methylation was a part of the regulatory mechanism for allelic silencing in allopolyploid fish (Matos et al., 2016). In a study with tilapia, Konstantinidis et al. (2021) showed tissue-specific differential methylation of genes involved in somatic growth, such as growth factors and their receptors, with hypomethylation in the muscle tissues. All these studies demonstrated the involvement of DNA methylation in regulation of fish growth. With Giant freshwater prawn, *Macrobrachium rosenbergii*, increased levels of genomic methylation were observed with the “iron prawn”, prawns with serious growth retardation (Jiang et al., 2020). This is similar to the situation in Japanese flounder, where increased methylation of MyoD and IGF genes are correlated with reduced growth (Huang et al., 2018).

### 3.3 Genetic and epigenetic regulation of disease resistance

Great efforts have been made to understand the molecular basis for disease resistance. Studies on genetic basis have focused on identification of QTL controlling disease resistance or susceptibility, and determination of causal genes (Table 2). A good example is the determination of resistance for the infectious pancreatic necrosis virus (IPNV) in Atlantic salmon (Houston et al., 2008; Houston et al., 2010; Moen et al., 2015; Pavelin et al., 2021). In a series of studies, these researchers identified a single QTL fully responsible for the disease resistance, and such information has been applied in the aquaculture industry to control IPVN. Interestingly, earlier studies seemed to indicate that a cellular receptor for the virus, cadherin 1, was the causal gene (Moen et al., 2015). However, further analysis of whole genome sequencing and functional annotation, knockout, and differential expression analysis of homozygous resistant and susceptible fish after infection allowed the identification of NEDD-8 activating enzyme 1 (nae1) as the causal gene (Pavelin et al., 2021). As summarized in a recent white paper (Abdelrahman et al., 2017), QTL studies have been conducted to identify genetic variants and genomic regions associated with disease resistance in various aquaculture species, including channel catfish, Atlantic salmon, rainbow trout, Asian seabass, and Japanese flounder, among many other aquaculture species (Table 2). Moreover, transcriptomic analyses after disease infection and stress challenges were conducted in aquaculture species (Qian et al., 2014; Jin et al., 2022), providing insights into differentially expressed genes, and their involved gene pathways. Genes important for immune response were identified in Atlantic salmon using RNA-Seq (Fu et al., 2022). With catfish, Jin et al. (2022) applied the bulk segregant analysis and RNA-Seq (BSR-Seq) to determine genes involved in QTLs important for ESC resistance. Such analysis allowed identification of potential candidate genes for ESC resistance. As summarized in Table 2, one of the major characteristics of disease resistance in aquaculture species is that just one or few genes control disease resistance in aquatic species. This has been demonstrated with viral diseases, as well as bacterial diseases.

**TABLE 2:** Some examples of genetic studies of disease resistance in several major aquaculture fish species.

Species	Diseases	QTL and candidate genes	References
Atlantic salmon	Infectious pancreatic necrosis (IPN), viral	A single QTL on chromosome 26 explains all the resistance, and NEDD-8 activating enzyme 1 was the candidate causal gene	Moen et al. (2009); Moen et al. (2015); Houston et al. (2010); Pavelin et al. (2021)
	Pancreas disease (PD), viral	QTL mapped to chromosome 3 and chromosome 7	Gonen et al. (2015); Hillestad et al. (2020a)
	Infectious salmon anemia (ISA), viral	QTL mapped to chromosome 15	Moen et al. (2007); Li et al. (2011); Gervais et al. (2021)
	Cardiomyopathy syndrome, viral	QTL mapped to chromosome 27	Hillestad and Moghadam, (2019); Hillestad et al. (2020b)
	Amoebic gill disease (AGD), parasitic	QTL mapped to various chromosomes 1, 2, 5, 4, 9, 13	Boison et al. (2019); Aslam et al. (2020)
	Sea lice, parasitic	QTL mapped to chromosomes 3, 18, 21	Robledo et al. (2019)
Rainbow trout	Bacterial coldwater disease	QTL for resistance was mapped to chromosome 19, 8 and 25	Wiens et al. (2013); Vallejo et al. (2014); Liu et al. (2015); Palti et al. (2015); Fraslin et al. (2019); Liu et al. (2022)
	Whirling disease	A single QTL was identified on chromosome 9 for resistance	Baerwald et al. (2011)
	Columnaris disease (CD), bacterial	Major QTL were mapped to chromosomes 3 and 5	Calboli et al. (2022); Fraslin et al. (2022)
		A major QTL was identified for resistance to rhabdovirus	Verrier et al. (2013)
	White spot disease (WSD), parasitic ciliate *Ichthyophthirius multifiliis*	Two QTL were identified on chromosome 16 and 17	Jaafar et al. (2020)
Common carp	RNA-Seq	DEGs related to CyHV-3 disease resistance were identified	Tadmor-Levi et al. (2019a)
Common carp	Cyprinid herpes virus disease	QTL mapped to chromosome 14, 30, 43, 44, and 46	Palaiokostas et al. (2018)
			Tadmor-Levi et al. (2019b)
Grass carp	Grass carp reovirus	Resistance against GCRV has high heritability	Huang et al. (2015)
Catfish	Enteric septicemia of catfish (ESC) disease, bacterial	QTL were mapped to chromosome 1, 12, and 16	Zhou et al. (2017); Shi et al. (2018); Tan et al. (2018b)
			Jin et al. (2022)
	Columnaris disease, bacterial	QTL mapped to linkage group 7, 12, and 14 in genomic hubs	Geng et al. (2015)
			Zhang et al. (2020)
	Aeromonas septicemia disease, bacterial	QTL mapped to linkage groups 2, 26, and 29	Wang et al. (2019)
Asian seabass	Viral nervous necrosis disease (VNN or NNV)	QTL and suggestive QTL were identified	Liu et al. (2016); Yang et al. (2020)
Gilthead sea bream	Pasteurellosis disease	Two significant QTL were identified	Massault et al. (2011)
Japanese flounder	Lymphocystis disease, viral	Marker-assisted selection	Fuji et al. (2007)
Turbot	Aeromonas disease, bacterial	QTLs were identified	Rodríguez-Ramilo et al. (2011)

In contrast to extensive genetic studies, studies of epigenetic regulation of disease resistance are still limited. In grass carp, hypermethylation of GC island upstream of the RIG-I gene in the susceptible fish led to reduced expression of RIG-I gene, which in turn accounted for the observed susceptibility for the grass carp reovirus (Shang et al., 2016). With brine shrimp (*Artemia franciscana*), biological control treatment with a plant-based phenolic compound resulted in transgenerational inherited increased resistance against *Vibrio parahaemolyticus*, the pathogen for acute hepatopancreatic necrosis (Roy et al., 2019, 2022), and DNA methylation was involved in the elevated expression of innate immune genes.

### 3.4 Genetic and epigenetic regulation of heat stress

Heat stress is increasingly a problem for aquatic organisms with the trend of climate change. Heat stress tolerance is particularly important for cold- and cool-water species. As such, much work has been conducted with salmonids. QTL for upper temperature tolerance (UTT) have been identified (Jackson et al., 1998; Perry et al., 2001; Somorjai et al., 2003). Genes associated with UTT have been identified; small heat shock proteins, along with hsp90, were found to be associated with UTT (Quinn et al., 2011). Strains were developed to have enhanced upper temperature tolerance for rainbow trout (Chen et al., 2015; Tan et al., 2016). Similarly, QTL for UTT were identified in turbot (Ma A. et al., 2021).

Genetic research of heat stress tolerance with warmwater fish is rare. While temperature may not be a major factor for survival with warmwater fish, the adverse impacts of high temperature on growth, disease resistance and sex reversal (see below) make it important to study heat stress tolerance with warmwater fish as well. Jin et al. (2017) identified three significant loci associated with tolerance to heat stress in channel catfish, a warmwater species. Genes included in these QTL regions included those involved in protein folding, protein degradation and protein synthesis, as well as those for iron transport and cytoskeletal reorganization.

Temperature is probably a single most frequent and most important environmental factor for poikilothermic animals such as fish. With just a few degrees of temperature change, thousands of genes are differentially expressed (e.g., Liu et al., 2013). The question is how such expression is regulated. Although the detailed mechanisms await to be elucidated, it is apparent that DNA methylation is intensely involved. Several studies have been conducted with epigenetic regulation of heat stress resistance. These studies provided strong evidence for intergenerational inheritance of acquired traits that were epigenetically regulated. With an *Artemia* model, Norouzitallab et al. (2014) conducted common garden experiments, where the *Artemia* was exposed to nonlethal heat shocks. The parental population was observed with increased expression of heat shock protein 70, and they are more tolerant to lethal heat stress, and more resistant against pathogenic *Vibrio campbellii*. Most interestingly, they found that the acquired phenotypic traits were transmitted to three successive generations without any additional exposure to heat stress. However, in this study, the measurement was levels of global DNA methylation and acetylated histones H3 and H4, not specific epigenetic marks (Norouzitallab et al., 2014). In a separate study, Robinson et al. (2019) demonstrated that early developmental stress can affect subsequent gene expression response to an acute stress in Atlantic salmon. Using reduced representation bisulfite sequencing, they found differences in methylation in the genomic neighborhood of the response genes, but the patterns of methylation was complicated (Robinson et al., 2019). Similarly, a study using zebrafish demonstrated complex interactions of temperature, DNA methylation and other environmental factors (Pierron et al., 2021). In that work, they found strong correlation of heat and methylation level of cyp19a1a gene with population masculinization.

High temperature stress may be a real threat to many aquatic species in the face of global climate change. Adaptive phenotypic response through epigenetic regulation may be particularly important for K-strategy species, where the species population are maintained at its maximal capacity as allowed by the environment, as demonstrated with winter skate (*Leucoraja ocellata*) (Lighten et al., 2016). Such adaptive responses are believed to be regulated by epigenetic regulation. DNA methyltransferase 3a was shown to mediate developmental thermal plasticity in zebrafish (Loughland et al., 2021) as its knockout led to decrease survival and increased deformities under cold temperatures. High temperature stress caused a significant increase of *de novo* DNA methyltransferase genes although it did not cause global cytosine methylation levels during reprogramming of DNA methylation (Dorts et al., 2016). There is a clear gender-specific response to temperature stress, as demonstrated by the work with Chinese tongue sole (*Cynoglossus semilaevis*), where approximately a quarter of the differentially expressed genes were shared among males, females, and pseudo-males (Wang et al., 2020). Although the literature is still limited at present, it is apparent that high temperature stress may have a fundamental impact on fish and shellfish species, affecting their growth, development, and sex phenotypes, mostly through the mechanisms of epigenetic regulation, especially with global climate change scenarios. With European seabass, increases of even 2°C in larvae significantly changed global DNA methylation and the expression of ecologically-relevant genes related to DNA methylation, stress response, muscle and organ formations (Anastasiadi et al., 2017). Similarly, with marine stickleback (*Gasterosteus aculeatus*), parental acclimation to ocean warming led to dynamic and temperature-sensitive re-programming throughout offspring development (Fellous et al., 2022).

### 3.5 Genetic and epigenetic regulation of tolerance to low oxygen

Aquatic organisms face frequent variations in dissolved oxygen in water. Under aquaculture conditions, hypoxia can be caused by natural phenomena (e.g., weather, temperature, or water flow rate), water pollution and eutrophication, high stocking density, and improper use of aeration. Aquatic species often are encountered with hypoxia (low oxygen) or even anoxia (no oxygen) environments. During the normal production cycle, aquaculture species experience great levels of variation in dissolved oxygen; even during a 24-h day and night shift, oxygen in the water vary greatly. In aquaculture ponds, oxygen levels are high during the sunny hours of the day and start to decline in the evening, related to the photosynthesis activities of algal species. As a result, aquaculture species must cope with such variations in dissolved oxygen concentrations. In the face of climate change and potential global warming, aquatic organisms are facing unprecedented challenges. While responses to high temperature can be different types of responses as compared to responses to hypoxia, temperature and oxygen conditions are much interwoven for aquatic organisms. Most often, high temperature could be related to reduced dissolved oxygen (Breitburg et al., 2018). In addition, exposure to hypoxia can also cause depression of the immune system in fish such as catfish, leading to increased susceptibility to diseases (Kvamme et al., 2013; Geng et al., 2014).

Much genetic and genomic research has been conducted to identify genes underlining low oxygen tolerance. With catfish, QTL analyses were conducted to localize low oxygen tolerance genes with both intra- and inter-specific systems (Wang et al., 2017c; Zhong et al., 2017). Selection signatures in the domestication processes that involved low oxygen conditions have been identified (Sun et al., 2014). Through RNA-Seq and gene expression studies, Yang et al. (2018) identified a large number of differentially expressed genes under hypoxic conditions in the swim bladder. Several gene pathways were involved in response to low oxygen including HIF signaling pathway, MAPK signaling pathway, PI3K/Akt/mTOR signaling pathway, Ras signaling pathway, and signaling by VEGF in the catfish swim bladder. A common set of genes important to both hypoxia and disease responses were identified, suggesting a common linkage of disease and hypoxia responses, such as claudin gene (Sun et al., 2015), CC chemokines (Fu et al., 2017a; 2017b), and their receptors (Fu et al., 2017c), Bcl-2 (Yuan et al., 2016), as well as hypoxia-specific responses such as hypoxia inducible factors 1 alpha (HIF-1) and hypoxia inducible factor inhibiting factor (FIH-1) (Geng et al., 2014). Similarly, hypoxia tolerance QTL have been identified from tilapia (Li et al., 2017), and in *Pelteobagrus vachelli* (Zhang et al., 2020). HIF-1 was found important for hypoxia in red swamp cray fish (Xu et al., 2022). With bighead catfish, Ma et al. (2021) identified 26 candidate genes involved in air-breathing development and function.

Good progress has been made in understanding of the involvement of DNA methylation in tolerance of hypoxia and anoxia. DNA methylation has regulatory functions in the Pacific oyster (*Crassostrea gigas*), particularly in gene families that have inducible expression, including those involved in the stress and environmental responses (Gavery and Roberts, 2010). Beemelmanns et al. (2021) used a set of biomarker genes for temperature stress (cirbp, serpinh1), oxidative stress (prdx6, ucp2), apoptosis (jund), and metabolism (pdk3), and uncovered distinct CpG methylation profiles under high temperature and low oxygen. With freshwater turtle, Wijenayake and Storey (2016) determined expression of four DNA methyltransferases, DNMT1, DNMT2, DNMT3A, and DNMT3B, and two methyl-binding domain proteins, MBD1 and MBD2, and found upregulated expression of these genes in the liver and white muscles under anoxic submergence of the organism, suggesting increased DNA methylation under hypoxic conditions. With rainbow trout, hypoxia treatment induced expression of BCL2 interacting protein 3 (bnip3) and its related genes. Such induced expression was found to be regulated by DNA methylation (Veron et al., 2018). Similarly, reduced levels of methylation in the promoter regions of STAT3 and VEGFA genes were found to be correlated with their increased expression under hypoxic conditions (Li et al., 2022a).

### 3.6 Genetic and epigenetic sex determination and regulation of sex differentiation

Teleost fish exhibit a tremendous level of diversity and plasticity in sex determination. Not only genotypes are important for sex determination, in many cases the environment, especially the temperature, can exert its effect on sex determination. Thus, genetic sex determination (GSD) and temperature-dependent sex determination (TSD) may be operating in the same or different species of lower vertebrates.

Extensive research has been conducted with sex determination and differentiation. The first sex determination gene in fish was identified from medaka, where DMY gene, a duplicate gene of DMRT1 on the Y chromosome, was identified as the sex determination gene in medaka fish *Oryzias latipes* (Matsuda et al., 2002). This gene was found as the sex determination gene in closely related medaka species of *O. curvinotus* (Table 3). However, in a different medaka species of *O. luzonensis*, GsdfY, the gonadal soma derived growth factor on the Y chromosome, was found to be the master sex determination gene, suggesting rapid evolution of master sex determination genes among teleost fish (Myosho et al., 2012).

**TABLE 3:** Sex determination genes in selected fish species.

Species	Sex determination gene	References
Medaka, *Oryzias latipes*, and *O. curvinotus*	DMY (duplicate of DMRT1 on Y chromosome)	Matsuda et al. (2002); Matsuda et al. (2003); Nanda et al. (2002); Zhang, (2004)
Medaka, *O. luzonensis*, Sablefish (*Anoplopoma fimbria*)	GsdfY (Gonadal soma derived growth factor on the Y chromosome)	Myosho et al. (2012)
		Herpin et al. (2021)
Rainbow trout, Atlantic salmon	Sdy (Sexually dimorphic on the Y-chromosome)	Yano et al. (2012); Yano et al. (2013)
Chinese tongue sole	DMRT1 (Doublesex and mab-3 related transcription factor 1)	Chen et al. (2014)
Nile tilapia	AMH (anti-Müllerian hormone)	Li et al. (2015); Cáceres et al. (2019)
Sebastes rockfish, Northern pike, *Odontesthes hatcheri*	AMH (Male-specific duplication of anti-Müllerian hormone)	Hattori et al. (2012); Pan et al. (2019); Song et al. (2021)
Fugu	Amhr2	Kamiya et al. (2012)
Seriola fishes	Hsd17b1 (17β-hydroxysteroid dehydrogenase 1)	Koyama et al. (2019)
Channel catfish	Epigenetically controlled allelic expression	Yang et al. (2022)

However, new master sex determination genes are generally recruited from genes involved in the downstream of the sex determination regulatory genetic network. These included DMRT1, Gsdf, AMH, Amhr2, and Hsd17b1 (Table 3). The only exception is Sdy, the master sex determination gene in rainbow trout and Atlantic salmon (Yano et al., 2012, 2013). Sdy is a duplicated immune-related gene that became integrated in the classical vertebrate sex differentiation cascade by interacting with the Forkhead box domain of the female-determining transcription factor, Foxl2 (Bertho et al., 2018). In the presence of Foxl2, SdY is translocated to the nucleus where the SdY:Foxl2 complex prevents activation of the aromatase (cyp19a1a) promoter, thereby disrupting the female differentiation pathway, consequently allowing testicular differentiation to proceed (Bertho et al., 2018). In channel catfish, we have identified an epigenetically marked locus within the sex determination region (SDR), where hypermethylation was found on the X chromosomes but hypomethylation was found on the Y chromosome, leading to differential expression of the sex determination gene (Yang et al., 2022). Similarly, with the Siamese fighting fish (*Betta splendens*), genetic sex is determined by an X/Y system with dmrt1 gene as the master sex determination gene, but the expression of the dmrt1 gene on the X chromosome is down-regulated by changes in DNA methylation caused by transposon-induced epigenetic silencing, where a transposon, drbx1, inserted into the fourth intron of the X-linked dmrt1 allele (Wang et al., 2022).

Extensive studies have been conducted with epigenetic regulation of sex determination and/or differentiation. These studies have focused on the following aspects of epigenetic regulation: 1) Sexual dimorphism in DNA methylation patterns; 2) Epigenetic regulation through DNA methylation of key genes involved in the sex determination regulatory genetic network and/or sex differentiation; and 3) Involvement of epigenetic regulation in temperature (or other factors)-induced sex reversal. Sexual dimorphism of methylation patterns was observed with several fish species. Some examples are summarized in Table 4. Detailed examination of the sex differences in methylation patterns differentiates the sexual methylation pattern dimorphism into two categories: 1) The difference is concentrated on sex chromosome, and 2) the differences are general. In this regard, there are many similarities between the situations of three spine stickleback (Metzger and Schulte, 2018) and channel catfish (Wang et al., 2022; Yang et al., 2022), where sex dimorphism in DNA methylation is concentrated on the sex chromosome. In both cases, hypermethylation was observed on the X chromosome and hypomethylation was observed on the Y chromosome. In channel catfish, such differential methylation was observed within the SDR, suggesting the importance of DNA methylation in sex determination and differentiation.

**TABLE 4:** Sexual dimorphism in patterns of DNA methylation.

Species	Major findings	References
Tilapia	Differential methylation patterns between males and females	Wan et al. (2016)
Threespine stickleback	Hypermethylation of X chromosome compared to Y chromosome	Metzger and Schulte, (2018)
Ussuri Catfish	Differential methylation patterns between males and females	Li et al. (2022b)
Channel catfish	Sex-specific epigenetically marked locus was identified in the sex determination region (SDR)	Yang et al. (2022)
Oyster	Methylomes are related to infertility of triploid oysters	Sun et al. (2022)

Many studies have focused on the relationship between expression of key genes involved in sex determination regulatory genetic network and DNA methylation. Table 5 shows many examples of such research. In general, key genes for female gonadal differentiation such as cyp19a are differentially expressed and differentially methylated, with higher expression and lower levels of methylation in females than in males. In contrast, key genes for male gonadal differentiation such as dmart1 are differentially expressed and differentially methylated, with higher expression and lower levels of methylation in males than in females. However, in most cases, only the promoter regions were under investigation, and the definition of the promoter vary among studies. In addition, the correlation is generally not linear. The traditional notion that DNA methylation suppress gene expression may have its limitations (see Section 2.4). Whatever the detailed mechanisms are, the ultimate difference it makes is the difference in gene expression, which is the core of the central dogma of molecular biology, but the key modification here is at the level of DNA methylation, i.e., epigenetic regulation, rather than being coded in DNA sequences.

**TABLE 5:** Differential DNA methylation of key genes involved in sex determination and/or differentiation.

Species	Major findings	References
European sea bass	Juvenile males have double the DNA methylation levels of females in the promoter of gonadal aromatase Cyp19a whose methylation is involved in temperature-dependent sex ratio shift	Navarro-Martín et al. (2011)
	Contrasting sex-specific methylation and temperature responses of cyp19a1a and dmrt1 genes	Anastasiadi et al. (2018)
Japanese flounder	High expression of dmrt1 and cyp19a in males and females, respectively, which are negatively correlated with methylation of their promoters	Wen et al. (2014)
Zebrafish	Differential CpG methylated in the brain	Chatterjee et al. (2016)
	Hypomethylation of esr1 promotor and overexpression in females; sex-specific patterns of transcription with dnmt1, dnmt3, and hdac1	Laing et al. (2018)
Nile tilapia	Cyp19a1a is important for high temperature-induced masculinization	Wang et al. (2017b)
	High expression and low methylation of cyp19a	Chen et al. (2018a)
*Hermaphrodite barramundi*	cyp19a1 and amh were more methylated in males, whereas dmrt1 and nr5a2 were more methylated in females	Domingos et al. (2018)
Chinese sea perch	Hypomethylated cyp19a1a promoter region in females	Chen et al. (2018b)
*Culter alburnus*	Dmrt1 gene hypomethylated and highly expressed in males	Jia et al. (2019)
Large yellow croaker	Key genes of sex determination are differentially methylated in males, females, and neomales	He et al. (2020a)
*Schizothorax kozlovi*	High expression and low methylation of dmrt1 in males	He et al. (2020b)
*Pelvicachromis pulcher*	Sex-specific hypomethylation and expression in females of copy A of cyp19a1 gene	Driscoll et al. (2020)
Orange-spotted grouper	Hypomethylated cyp19a1a promoter region in females	Guo et al. (2021)
Olive flounder	Global methylation was higher in testis than in ovary	Liu et al. (2021)

From the studies conducted to date (with some examples shown in Table 6), it is apparent that DNA methylation is involved in the temperature-dependent sex determination. However, high temperature apparently had opposite sex ratio shift with different species. In zebrafish, tilapia, and half-smooth tongue sole, high temperature induced masculinization, while high temperature induced feminization in channel catfish. These systems, therefore, provide a great comparative platform to determine the relationship of high temperature, DNA methylation, gene expression and phenotypic sex differentiation.

**TABLE 6:** Methylation/demethylation of specific genes and their involvement in sex reversal/sex ratio shift.

Species	Major findings	References
Tilapia	Upregulation of CYP11B2 and DMRT1 during high temperature-induced masculinization	Li et al. (2014)
	High temperature induced sex reversal to males is accompanied with higher methylation levels in the gonads	Sun et al. (2016)
Half-smooth tongue sole	Heat induced sex reversal into pseudomales are regulated by DNA methylation of key genes in sex determination pathway, and transgenerational inheritance after heat treatment was observed	Shao et al. (2014)
Zebrafish	Treatment of DNA methyltransferase inhibitor 5-Aza-dC feminizes zebrafish, with permanent alterations of gonadal transcriptome, with increased expression of key genes of female gonadal development	Ribas et al. (2017)
	High temperature induced sex shift toward males of parental families and F1 fish, but not F2 fish. Methylation was lower in F1, but not in P and F2 fish	Valdivieso et al. (2020)
	Sox9b and esr1 are down-regulated in high temperature-induced masculinization	Han et al. (2021b)
Channel catfish	Key genes for female gonadal development and male gonadal development were upregulated, and down-regulated, respectively, during hormone-induced feminization, but the methylation patterns were complex. These are independent from the sex determination region. High temperature induces sex reversal to females	Wang et al. (2022)
		Zhou et al., unpublished data

## 4 Histone modifications and their regulation of genome expression

There are numerous types of histone modifications, but several types of histone modifications are better understood as to how they are related to genome expression. These include histone methylation, acetylation and phosphorylation (Zhang et al., 2021). Histone methylation, usually at the lysine (K) residues of histone H3 and H4, are best understood in their roles for regulation of gene expression. The methylation is catalyzed by histone methyltransferase, which uses S-adenosyl methionine as the substrate to transfer methyl groups onto the lysine residues of histones. The lysine residues of histones can be mono-, di-, and tri-methylated to act as the active or repressive marks of gene expression. Gene activation has been correlated with H3K4, H3K36, and H3K79 methylation, while H3K9, H3K27, and H4K20 are known as repressive marks that are usually associated with the silenced gene expression and heterochromatin (Zhang et al., 2021). It is important to note that multiple histone modifications may occur, and they collectively affect genome expression. Table 7 summarizes the general pattern of histone modifications and their correlated gene expression.

**TABLE 7:** Patterns of histone modification and regulation of gene expression.

Histone modification	Effect on gene expression	References
H3K4me3	Active promoters	Zhang et al. (2016)
H3K9me3	Gene repression and heterochromatin formation	Bannister et al. (2001)
H3K27me3	Gene repression	Zhang et al. (2015)
H3K36me3	Active genes	Oqani et al. (2019)
H3K79me3	Active transcription	Ooga et al. (2008)
H4K20 me1	Repressive hallmark	Sato et al. (2016)
H3K27ac	Active gene enhancers, often co-exist with H3K4me3	Kang et al. (2021)

Histone modifications can be most efficiently studied by chromatin immunoprecipitation with high-throughput sequencing (ChIP-Seq). ChIP-Seq is a powerful technology to locate regulatory elements, as indicated by association of histone modifications such as H3K4me3 with actively transcribed gene promoters, and H3K27ac with enhancers. It is also very useful to locate sites involved in gene silencing, as indicated by association of some histone modifications with repression of gene expression such as H3K9me3 and H3K27me3 (Table 7). ChIP-Seq has been well demonstrated with human and mouse research with over a dozen different histone modifications being assayed, but less so with agricultural animals. However, as histone proteins are so highly conserved, the antibodies for mammalian species should, for the most part, work for various fish species.

Among teleost fish, ChIP-Seq has been primarily conducted in model systems. For example, Zhu et al. (2019) studied histone modification patterns with H3K4m3 and H3K27me3 early during development. In zebrafish, ChIP-Seq after infection of spring viremia of carp virus (SVCV) allowed identification of key immune genes of interferon signalling pathway and c-reactive protein genes, and demonstrated the importance of epigenetic modifications in response to viral infections (Medina-Gali et al., 2018). As a proof of concept, ChIP-Seq was conducted with tilapia (Kratochwil and Meyer, 2015). With common carp, increased H4K12ac was found to be associated with aging oocytes (Waghmare et al., 2021).

## 5 Intergenerational and transgenerational epigenetic inheritance

One of the most interesting questions is if the environmentally induced epigenetic changes and their regulated gene expression are inherited. For this discussion, we would like to differentiate intergenerational and transgenerational epigenetic inheritance. Intergenerational epigenetic inheritance refers to inheritance of epigenetic marks and/or their related phenotypes from one generation (F_0_) to the next (F_1_), while transgenerational epigenetic inheritance refers to the passage of information from grandparents to grandchild (F_2_) or later generations if paternal grandparent was exposed or F_3_ if maternal grandparent was exposed (Skinner and Guerrero-Bosagna, 2009; Lacal and Ventura, 2018).

Like the situation with mammalian species, where intergenerational epigenetic inheritance is not uncommon, but transgenerational epigenetic inheritance has not been fully demonstrated (For a review, see Cavalli and Heard, 2019), most studies with aquatic species have clearly supported intergenerational epigenetic inheritance. For example, with Atlantic salmon, captive rearing of adult caused intergenerational differential methylation in F1 (Wellband et al., 2021). However, it is not concretely solid with transgenerational epigenetic inheritance. Some examples of intergenerational and transgenerational epigenetic studies are provided in Table 8. Most of these studies were conducted with model species zebrafish, or microcrustacean *Daphnia* or *Artemia* species.

**TABLE 8:** Examples of studies of intergenerational and transgenerational epigenetic inheritance.

Species	Treatment	Epigenetic marks or phenotypic analysis		References
Half-smooth tongue sole	High temperature induced sex reversal to pseudomales	Pseudomales produced F_1_ pseudomales without temperature treatment	Intergenerational	Shao et al. (2014)
Zebrafish	Crude oil containing diet	Global methylation of F_1_ was decreased as exposed parents	Intergenerational	Bautista et al. (2020)
Atlantic salmon	Captivity	Differential methylation in F1 with exposed parents	Intergenerational	Wellband et al. (2021)
Pipefish	Heat-killed bacteria	Expression of genes involved in DNA methylation	Intergenerational and transgenerational	Beemelmanns and Roth, (2017)
Zebrafish	benzo[a]pyrene exposure of F0 fish	F2 fish exhibited altered phenotypes	Transgenerational	Knecht et al. (2017)
Zebrafish	Mercury exposure of F_0_	F_2_ fish exhibited altered phenotypes	Transgenerational	Carvan et al. (2017)
Zebrafish	Permethrin exposure of F_0_	F_2_ fish exhibited altered phenotypes	Transgenerational	
Zebrafish	F_0_ exposure to mono(2-ethylhexyl) phthalate and 5-azacytidine	Differential methylation in F_1_ and F_2_	Intergenerational and transgenerational	Kamstra et al. (2017)
Medaka	F_0_ exposure to bisphenol A and 17α-ethinylestradiol	Changed gene expression in F_2_	Transgenerational	Thayil et al. (2020)
Atlantic molly	Exposure of F_0_ fish to hydrogen sulfide	80% overlap of epigenetic profiles of F_2_ of treated F_0_ fish	Transgenerational	Kelley et al. (2021)
*Artemia franciscana*	Phloroglucinol Treatment	Elevated methylation was observed in F_1_, and in cysts in F_2_ but not in F_2_ nor F_3_ of juveniles	Intergenerational and perhaps Transgenerational	Roy et al. (2019)
	*Vibrio* challenge or injection	Treated progenies had transgenerational immune memory		Roy et al. (2022)
*Daphnia magna *	Zinc exposure	Significant reduction of cytosine methylation in F_1_ but not in F_2_	Intergenerational	Vandegehuchte et al. (2009); Vandegehuchte et al. (2010a)
*Daphnia magna *	Exposure to 5-azacytidine		Transgenerational	Vandegehuchte et al. (2010b)
*Daphnia magna *	Chronic external γ-irradiation	Changes of DNA methylation were observed in F_2_ and F_3_ animals, but not very specific	Transgenerational	Trijau et al. (2018)
*Daphnia magna *	High salinity	Differential methylation of a set of specific genes were observed in exposed F0 and unexposed F_1_, F_2_, and F_3_ animals	Transgenerational	Jeremias et al. (2018)

As shown in Table 8, most of these studies were conducted for just two generations, and in some cases for three generations. In addition, in many of these cases, the measurements were general performance traits, rather than specific molecular patterns. Even when DNA methylation was determined, demonstration of transgenerational epigenetic inheritance with specific patterns of DNA methylation is very limited. Perhaps a best example is from a recent study with Atlantic molly (*Poecilia mexicana*), where the epigenetic alterations under hydrogen sulfide are inherited to laboratory-reared, non-exposed F2 fish (Kelley et al., 2021). When epigenetic changes in response to hydrogen sulfide were examined in red blood cells, there was over 80% overlap in differentially methylated regions (DMRs) across generations, suggesting that DMRs have stable generational inheritance in the absence of the sulfidic environment.

With *D. magna*, treatment with 5-azacytidine reduced the level of DNA methylation, and such reduced level of methylation was stably transferred to two subsequent non-exposed generations (Vandegehuchte et al., 2010a). A reduced level of methylation was observed after exposure to zinc, and such patterns of reduced methylation and correlated gene expression was observed in F_1_ animals, but not in F_2_ animals (Vandegehuchte et al., 2010b), suggesting intergenerational epigenetic inheritance. γ-irradiation-induced DNA methylation patterns were found to be inheritable, as common methylation patterns were observed from unexposed F_2_ and F_3_ individuals, but specific patterns of methylation were not reported (Trijau et al., 2018). High levels of salinity led to hypomethylation of treated F_0_ animals, and such hypomethylation was transgenerationally inherited to three consequent nonexposed generations (F_1_, F_2_, and F_3_) (Jeremias et al., 2018).

With brine shrimp (*Artemia franciscana*), phloroglucinol treatment significantly enhanced the expression of a core set of innate immune genes and resistance against bacterial infections of *Vibrio parahaemolyticus* and *V. harveyi*. Such enhanced resistance was observed in their progeny in three subsequent generations (Roy et al., 2019, 2022). In a separate study, Norouzitallab et al. (2014) exposed *Artemia* to nonlethal heat shocks, which increased the expression of Hsp70. The treated *Artemia* exhibited increased levels of tolerance for lethal heat stress, and resistance against pathogenic *Vibrio campbellii*. These acquired phenotypic traits were transmitted to three successive generations, none of which were exposed to the parental stressor. This transgenerational inheritance of the acquired traits was associated with altered levels of global DNA methylation and acetylated histones H3 and H4 in the heat-shocked group compared to the control group, where both the parental and successive generations were reared at standard temperature. These results indicated that epigenetic mechanisms, such as global DNA methylation and histones H3 and H4 acetylation, have particular dynamics that are crucial in the heritability of the acquired adaptive phenotypic traits across generations.

## 6 Future perspectives

Although the current literature of epigenetic regulation of aquaculture traits is limited, it is apparent that epigenetic regulation is involved in most, if not all, traits important for aquaculture. This is because ultimately what is important is the qualitative and quantitative expression of genes; and DNA methylation regulates both. This importance is reflected in normal processes of the life cycle, and in response to the ever-changing environment. Under normal conditions, aquaculture organisms are composed of various tissues, with nearly all of them having identical genomic sequences, and yet they can maintain their respective functions over the lifetime of the organism. This challenge is met mostly through epigenetic regulation including chromatin accessibility, DNA and histone modifications, activity of RNA, and protein factors interacting with the genome (Nakamura et al., 2021). In terms of development and differentiation, much needs to be learned from aquaculture species because finfish and reptiles are lower vertebrates where many of the developmental processes are conserved with those reported from higher vertebrates, but they show greater levels of plasticity that can well be epigenetically regulated. Therefore, much can be learned from studies with aquaculture species.

Although DNA methylation is perhaps the most studied of epigenetic regulation of aquaculture performance and production traits, the exact mechanisms of epigenetic regulation requires great attention. The studies of histone modifications and their relations with aquaculture performance and production traits are so limited to date, but this apparently demands research efforts, especially in understanding of transcriptional activation and inactivation. Finally, the regulation by ncRNAs is extremely important and likely is involved in expression of aquaculture performance and production traits; although this review intentionally left this out because systematic knowledge of epigenetic regulation of aquaculture traits is very limited at this time, but the importance of research in these areas cannot be overstated.

In addition to understanding epigenetic regulation, precise epigenome editing technologies have become available (for reviews, see Laufer and Singh, 2015; Goell and Hilton, 2021; Nakamura et al., 2021). Applications of epigenome editing technologies will deepen our understanding of epigenetic regulation from the level of correlation to the level of establishing causal effect relationships between epigenome modifications and performance traits. More directly, epigenome editing will also allow “engineering” of desired traits, based on information of epigenetic regulation.

## References

[B1] Abdelrahman H. ElHady M. Alcivar-Warren A. Allen S. Al-Tobasei R. Bao L. (2017). Aquaculture genomics, genetics and breeding in the United States: Current status, challenges, and priorities for future research. BMC Genomics 18 (1), 191. 10.1186/s12864-017-3557-1 28219347 PMC5319170

[B2] Alharbi A. B. Schmitz U. Bailey C. G. Rasko J. E. J. (2021). CTCF as a regulator of alternative splicing: New tricks for an old player. Nucleic Acids Res. 49 (14), 7825–7838. 10.1093/nar/gkab520 34181707 PMC8373115

[B3] Anastasiadi D. Díaz N. Piferrer F. (2017). Small ocean temperature increases elicit stage-dependent changes in DNA methylation and gene expression in a fish, the European sea bass. Sci. Rep. 7 (1), 12401. 10.1038/s41598-017-10861-6 28963513 PMC5622125

[B4] Anastasiadi D. Vandeputte M. Sánchez-Baizán N. Allal F. Piferrer F. (2018). Dynamic epimarks in sex-related genes predict gonad phenotype in the European sea bass, a fish with mixed genetic and environmental sex determination. Epigenetics 13 (9), 988–1011. 10.1080/15592294.2018.1529504 30265213 PMC6284782

[B5] Aslam M. L. Boison S. A. Lillehammer M. Norris A. Gjerde B. (2020). Genome-wide association mapping and accuracy of predictions for amoebic gill disease in Atlantic salmon (*Salmo salar*). Sci. Rep. 10 (1), 6435. 10.1038/s41598-020-63423-8 32296114 PMC7160127

[B6] Asselman J. De Coninck D. I. Pfrender M. E. De Schamphelaere K. A. (2016). Gene body methylation patterns in Daphnia are associated with gene family size. Genome Biol. Evol. 8 (4), 1185–1196. 10.1093/gbe/evw069 27017526 PMC4860698

[B7] Baerwald M. R. Petersen J. L. Hedrick R. P. Schisler G. J. May B. (2011). A major effect quantitative trait locus for whirling disease resistance identified in rainbow trout (*Oncorhynchus mykiss*). Heredity 106 (6), 920–926. 10.1038/hdy.2010.137 21048672 PMC3186244

[B8] Balasubramanian S. Raghunath A. Perumal E. (2019). Role of epigenetics in zebrafish development. Gene 718, 144049. 10.1016/j.gene.2019.144049 31430520

[B9] Bannister A. J. Zegerman P. Partridge J. F. Miska E. A. Thomas J. O. Allshire R. C. (2001). Selective recognition of methylated lysine 9 on histone H3 by the HP1 chromo domain. Nature 410 (6824), 120–124. 10.1038/35065138 11242054

[B10] Bautista N. M. Crespel A. Crossley J. Padilla P. Burggren W. (2020). Parental transgenerational epigenetic inheritance related to dietary crude oil exposure in *Danio rerio* . J. Exp. Biol. 223 (16), jeb222224. 10.1242/jeb.222224 32620709

[B11] Beemelmanns A. Ribas L. Anastasiadi D. Moraleda-Prados J. Zanuzzo F. S. Rise M. L. (2021). DNA methylation dynamics in Atlantic salmon (*Salmo salar*) challenged with high temperature and moderate hypoxia. Front. Mar. Sci. 7, 1076. 10.3389/fmars.2020.604878

[B12] Beemelmanns A. Roth O. (2017). Grandparental immune priming in the pipefish *Syngnathus typhle* . BMC Evol. Biol. 17 (1), 44. 10.1186/s12862-017-0885-3 28173760 PMC5297188

[B13] Berthelot C. Brunet F. Chalopin D. Juanchich A. Bernard M. Noël B. (2014). The rainbow trout genome provides novel insights into evolution after whole-genome duplication in vertebrates. Nat. Commun. 5, 3657. 10.1038/ncomms4657 24755649 PMC4071752

[B14] Bertho S. Herpin A. Branthonne A. Jouanno E. Yano A. Nicol B. (2018). The unusual rainbow trout sex determination gene hijacked the canonical vertebrate gonadal differentiation pathway. Proc. Natl. Acad. Sci. U. S. A. 115 (50), 12781–12786. 10.1073/pnas.1803826115 30463951 PMC6294932

[B15] Boison S. A. Gjerde B. Hillestad B. Makvandi-Nejad S. Moghadam H. K. (2019). Genomic and transcriptomic analysis of amoebic gill disease resistance in atlantic salmon (*Salmo salar* L.). Front. Genet. 10, 68. 10.3389/fgene.2019.00068 30873203 PMC6400892

[B16] Boyd C. E. (2015). “Overview of aquaculture feeds: Global impacts of ingredient use,” in Feed and feeding practices in aquaculture. Editor Davis D. A. (Oxford: Woodhead Publishing), 3–25.

[B17] Breitburg D. Levin L. A. Oschlies A. Grégoire M. Chavez F. P. Conley D. J. (2018). Declining oxygen in the global ocean and coastal waters. Science 359 (6371), eaam7240. 10.1126/science.aam7240 29301986

[B18] Cáceres G. López M. E. Cádiz M. I. Yoshida G. M. Jedlicki A. Palma-Véjares R. (2019)., 9. PMC6778786, 3213–3223. 10.1534/g3.119.400297 Fine mapping using whole-genome sequencing confirms anti-müllerian hormone as a major gene for sex determination in farmed nile Tilapia (*Oreochromis niloticus* L). G3 (bethesda) 10 G3 31416805 PMC6778786

[B19] Calboli F. C. F. Koskinen H. Nousianen A. Fraslin C. Houston R. D. Kause A. (2022). Conserved QTL and chromosomal inversion affect resistance to columnaris disease in 2 rainbow trout (*Oncorhyncus mykiss*) populations. G3 (Bethesda) 12 (8), jkac137. 10.1093/g3journal/jkac137 35666190 PMC9339330

[B20] Carvan M. J. 3rd Kalluvila T. A. Klingler R. H. Larson J. K. Pickens M. Mora-Zamorano F. X. (2017). Mercury-induced epigenetic transgenerational inheritance of abnormal neurobehavior is correlated with sperm epimutations in zebrafish. PLoS One 12 (5), e0176155. 10.1371/journal.pone.0176155 28464002 PMC5413066

[B21] Cavalli G. Heard E. (2019). Advances in epigenetics link genetics to the environment and disease. Nature 571 (7766), 489–499. 10.1038/s41586-019-1411-0 31341302

[B22] Chatterjee A. Lagisz M. Rodger E. J. Zhen L. Stockwell P. A. Duncan E. J. (2016). Sex differences in DNA methylation and expression in zebrafish brain: A test of an extended 'male sex drive' hypothesis. Gene 590 (2), 307–316. 10.1016/j.gene.2016.05.042 27259666

[B23] Chen S. Zhang G. Shao C. Huang Q. Liu G. Zhang P. (2014). Whole-genome sequence of a flatfish provides insights into ZW sex chromosome evolution and adaptation to a benthic lifestyle. Nat. Genet. 46 (3), 253–260. 10.1038/ng.2890 24487278

[B24] Chen X. He Y. Wang Z. Li J. (2018a). Expression and DNA methylation analysis of cyp19a1a in Chinese sea perch *Lateolabrax maculatus* . Comp. Biochem. Physiol. B Biochem. Mol. Biol. 226, 85–90. 10.1016/j.cbpb.2018.07.008 30099195

[B25] Chen X. Zhao Y. He Y. Zhao J. (2018b). Methylation pattern polymorphism of cyp19a in nile Tilapia and hybrids. Open Life Sci. 13, 327–334. 10.1515/biol-2018-0040 33817100 PMC7874709

[B26] Chen Z. Omori Y. Koren S. Shirokiya T. Kuroda T. Miyamoto A. (2019). Phillippy AM; NISC Comparative Sequencing Program, Mullikin JC, Burgess SM. De novo assembly of the goldfish (*Carassius auratus*) genome and the evolution of genes after whole-genome duplication. Sci. Adv. 5 (6), eaav0547. 10.1126/sciadv.aav0547 31249862 PMC6594761

[B27] Chen Z. Snow M. Lawrence C. S. Church A. R. Narum S. R. Devlin R. H. (2015). Selection for upper thermal tolerance in rainbow trout (*Oncorhynchus mykiss* Walbaum). J. Exp. Biol. 218 (5), 803–812. 10.1242/jeb.113993 25573825

[B28] Conte M. A. Gammerdinger W. J. Bartie K. L. Penman D. J. Kocher T. D. (2017). A high quality assembly of the Nile Tilapia (*Oreochromis niloticus*) genome reveals the structure of two sex determination regions. BMC Genomics 18 (1), 341. 10.1186/s12864-017-3723-5 28464822 PMC5414186

[B29] Cross S. H. Meehan R. R. Nan X. Bird A. (1997). A component of the transcriptional repressor MeCP1 shares a motif with DNA methyltransferase and HRX proteins. Nat. Genet. 16 (3), 256–259. 10.1038/ng0797-256 9207790

[B30] Dasmahapatra A. K. Khan I. A. (2015). DNA methyltransferase expressions in Japanese rice fish (*Oryzias latipes*) embryogenesis is developmentally regulated and modulated by ethanol and 5-azacytidine. Comp. Biochem. Physiol. C. Toxicol. Pharmacol., 176–177. 10.1016/j.cbpc.2015.07.002 26183885

[B31] Domingos J. A. Budd A. M. Banh Q. Q. Goldsbury J. A. Zenger K. R. Jerry D. R. (2018). Sex-specific dmrt1 and cyp19a1 methylation and alternative splicing in gonads of the protandrous hermaphrodite barramundi. PLoS One 13 (9), e0204182. 10.1371/journal.pone.0204182 30226860 PMC6143260

[B32] Dorts J. Falisse E. Schoofs E. Flamion E. Kestemont P. Silvestre F. (2016). DNA methyltransferases and stress-related genes expression in zebrafish larvae after exposure to heat and copper during reprogramming of DNA methylation. Sci. Rep. 6, 34254. 10.1038/srep34254 27731414 PMC5059630

[B33] Driscoll R. M. H. Faber-Hammond J. J. O'Rourke C. F. Hurd P. L. Renn S. C. P. (2020). Epigenetic regulation of gonadal and brain aromatase expression in a cichlid fish with environmental sex determination. Gen. Comp. Endocrinol. 296, 113538. 10.1016/j.ygcen.2020.113538 32585214

[B34] Eckersley-Maslin M. A. Alda-Catalinas C. Reik W. (2018). Dynamics of the epigenetic landscape during the maternal-to-zygotic transition. Nat. Rev. Mol. Cell Biol. 19 (7), 436–450. 10.1038/s41580-018-0008-z 29686419

[B35] Fellous A. Wegner K. M. John U. Mark F. C. Shama L. N. S. (2022). Windows of opportunity: Ocean warming shapes temperature-sensitive epigenetic reprogramming and gene expression across gametogenesis and embryogenesis in marine stickleback. Glob. Chang. Biol. 28 (1), 54–71. 10.1111/gcb.15942 34669228

[B36] Feng S. Cokus S. J. Zhang X. Chen P. Y. Bostick M. Goll M. G. (2010). Conservation and divergence of methylation patterning in plants and animals. Proc. Natl. Acad. Sci. U. S. A. 107 (19), 8689–8694. 10.1073/pnas.1002720107 20395551 PMC2889301

[B37] Fneich S. Dheilly N. Adema C. Rognon A. Reichelt M. Bulla J. (2013). 5-methyl-cytosine and 5-hydroxy-methyl-cytosine in the genome of *Biomphalaria glabrata*, a snail intermediate host of *Schistosoma mansoni* . Parasit. Vectors 6, 167. 10.1186/1756-3305-6-167 23742053 PMC3681652

[B38] Fraslin C. Brard-Fudulea S. D'Ambrosio J. Bestin A. Charles M. Haffray P. (2019). Rainbow trout resistance to bacterial cold water disease: Two new quantitative trait loci identified after a natural disease outbreak on a French farm. Anim. Genet. 50 (3), 293–297. 10.1111/age.12777 30883847

[B39] Fraslin C. Koskinen H. Nousianen A. Houston R. D. Kause A. (2022). Genome-wide association and genomic prediction of resistance to *Flavobacterium columnare* in a farmed rainbow trout population. Aquaculture 557, 738332. 10.1016/j.aquaculture.2022.738332

[B40] Fu Q. Yang Y. Li C. Zeng Q. Zhou T. Li N. (2017a). The chemokinome superfamily: II. The 64 CC chemokines in channel catfish and their involvement in disease and hypoxia responses. Dev. Comp. Immunol. 73, 97–108. 10.1016/j.dci.2017.03.012 28322933

[B41] Fu Q. Yang Y. Li C. Zeng Q. Zhou T. Li N. (2017b). The CC and CXC chemokine receptors in channel catfish (*Ictalurus punctatus*) and their involvement in disease and hypoxia responses. Dev. Comp. Immunol. 77, 241–251. 10.1016/j.dci.2017.08.012 28842182

[B42] Fu Q. Zeng Q. Li Y. Yang Y. Li C. Liu S. (2017c). The chemokinome superfamily in channel catfish: I. CXC subfamily and their involvement in disease defense and hypoxia responses. Fish. Shellfish Immunol. 60, 380–390. 10.1016/j.fsi.2016.12.004 27919758

[B43] Fu X. Zhang C. Zhang Y. (2020). Epigenetic regulation of mouse preimplantation embryo development. Curr. Opin. Genet. Dev. 64, 13–20. 10.1016/j.gde.2020.05.015 32563750 PMC7641911

[B44] Fuji K. Hasegawa O. Honda K. Kumasaka K. Sakamoto T. Okamoto N. (2007). Marker-assisted breeding of a lymphocystis disease-resistant Japanese flounder (*Paralichthys olivaceus*). Aquaculture 272 (1-4), 291–295. 10.1016/j.aquaculture.2007.07.210

[B45] Gao H. Bu Y. Wu Q. Wang X. Chang N. Lei L. (2015). Mecp2 regulates neural cell differentiation by suppressing the Id1 to Her2 axis in zebrafish. J. Cell Sci. 128 (12), 2340–2350. 10.1242/jcs.167874 25948585

[B46] Gao Z. You X. Zhang X. Chen J. Xu T. Huang Y. (2021). A chromosome-level genome assembly of the striped catfish (*Pangasianodon hypophthalmus*). Genomics 113 (5), 3349–3356. 10.1016/j.ygeno.2021.07.026 34343676

[B47] Gavery M. R. Roberts S. B. (2014). A context dependent role for DNA methylation in bivalves. Brief. Funct. Genomics 13 (3), 217–222. 10.1093/bfgp/elt054 24397979

[B48] Gavery M. R. Roberts S. B. (2017). Epigenetic considerations in aquaculture. PeerJ 5, e4147. 10.7717/peerj.4147 29230373 PMC5723431

[B49] Gavery M. R. Roberts S. B. (2010). DNA methylation patterns provide insight into epigenetic regulation in the Pacific oyster (*Crassostrea gigas*). BMC Genomics 11 (1), 483. 10.1186/1471-2164-11-483 20799955 PMC2996979

[B50] Geng X. Feng J. Liu S. Wang Y. Arias C. Liu Z. (2014). Transcriptional regulation of hypoxia inducible factors alpha (HIF-α) and their inhibiting factor (FIH-1) of channel catfish (*Ictalurus punctatus*) under hypoxia. Comp. Biochem. Physiol. B Biochem. Mol. Biol. 169, 38–50. 10.1016/j.cbpb.2013.12.007 24384398

[B51] Geng X. Sha J. Liu S. Bao L. Zhang J. Wang R. (2015). A genome-wide association study in catfish reveals the presence of functional hubs of related genes within QTLs for columnaris disease resistance. BMC Genomics 16 (1), 196. 10.1186/s12864-015-1409-4 25888203 PMC4372039

[B52] Gervais O. Barria A. Papadopoulou A. Gratacap R. L. Hillestad B. Tinch A. E. (2021). Exploring genetic resistance to infectious salmon anaemia virus in Atlantic salmon by genome-wide association and RNA sequencing. BMC Genomics 22 (1), 345. 10.1186/s12864-021-07671-6 33985436 PMC8117317

[B54] Gjedrem T. (2012). Genetic improvement for the development of efficient global aquaculture: A personal opinion review. Aquaculture 344-349, 12–22. 10.1016/j.aquaculture.2012.03.003

[B55] Goell J. H. Hilton I. B. (2021). CRISPR/Cas-Based epigenome editing: Advances, applications, and clinical utility. Trends Biotechnol. 39 (7), 678–691. 10.1016/j.tibtech.2020.10.012 33972106

[B56] Goll M. G. Bestor T. H. (2005). Eukaryotic cytosine methyltransferases. Annu. Rev. Biochem. 74, 481–514. 10.1146/annurev.biochem.74.010904.153721 15952895

[B57] Gómez-Chiarri M. Warren W. C. Guo X. Proestou D. (2015). Developing tools for the study of molluscan immunity: The sequencing of the genome of the eastern oyster, *Crassostrea virginica* . Fish. Shellfish Immunol. 46 (1), 2–4. 10.1016/j.fsi.2015.05.004 25982405

[B58] Gonen S. Baranski M. Thorland I. Norris A. Grove H. Arnesen P. (2015). Mapping and validation of a major QTL affecting resistance to pancreas disease (*Salmonid alphavirus*) in Atlantic salmon (*Salmo salar*). Hered. (Edinb). 115 (5), 405–414. 10.1038/hdy.2015.37 PMC4611234 25990876

[B59] Gong G. Dan C. Xiao S. Guo W. Huang P. Xiong Y. (2018). Chromosomal-level assembly of yellow catfish genome using third-generation DNA sequencing and Hi-C analysis. Gigascience 7 (11), giy120. 10.1093/gigascience/giy120 PMC6228179 30256939

[B60] Granada L. Lemos M. F. Cabral H. N. Bossier P. Novais S. C. (2018). Epigenetics in aquaculture – The last frontier. Rev. Aquac. 10, 994–1013. 10.1111/raq.12219

[B61] Guo C. Y. Tseng P. W. Hwang J. S. Wu G. C. Chang C. F. (2021). Potential role of DNA methylation of cyp19a1a promoter during sex change in protogynous orange-spotted grouper, *Epinephelus coioides* . Gen. Comp. Endocrinol. 311, 113840. 10.1016/j.ygcen.2021.113840 34216589

[B62] Han J. Hu Y. Qi Y. Yuan C. Naeem S. Huang D. (2021b). High temperature induced masculinization of zebrafish by down-regulation of sox9b and esr1 via DNA methylation. J. Environ. Sci. 107, 160–170. 10.1016/j.jes.2021.01.032 34412779

[B63] Han L. Sun Y. Cao Y. Gao P. Quan Z. Chang Y. (2021a). Analysis of the gene transcription patterns and DNA methylation characteristics of triploid sea cucumbers (*Apostichopus japonicus*). Sci. Rep. 11 (1), 7564. 10.1038/s41598-021-87278-9 33828212 PMC8027599

[B64] Harris C. J. Scheibe M. Wongpalee S. P. Liu W. Cornett E. M. Vaughan R. M. (2018). A DNA methylation reader complex that enhances gene transcription. Science 362 (6419), 1182–1186. 10.1126/science.aar7854 30523112 PMC6353633

[B65] Hattori R. S. Murai Y. Oura M. Masuda S. Majhi S. K. Sakamoto T. (2012). A Y-linked anti-Müllerian hormone duplication takes over a critical role in sex determination. Proc. Natl. Acad. Sci. U. S. A. 109 (8), 2955–2959. 10.1073/pnas.1018392109 22323585 PMC3286941

[B66] He A. Li W. Wang W. Ye K. Gong S. Wang Z. (2020a). Analysis of DNA methylation differences in gonads of the large yellow croaker. Gene 749, 144754. 10.1016/j.gene.2020.144754 32376450

[B67] He Y. Wu X. Zhu Y. Yang D. (2020b). Expression profiles of dmrt1 in *Schizothorax kozlovi*, and their relation to CpG methylation of its promoter and temperature. Zool. Sci. 37 (2), 140–147. 10.2108/zs190054 32282145

[B68] He Y. F. Li B. Z. Li Z. Liu P. Wang Y. Tang Q. (2011). Tet-mediated formation of 5-carboxylcytosine and its excision by TDG in mammalian DNA. Science 333 (6047), 1303–1307. 10.1126/science.1210944 21817016 PMC3462231

[B69] Hearn J. Plenderleith F. Little T. J. (2021). DNA methylation differs extensively between strains of the same geographical origin and changes with age in *Daphnia magna* . Epigenetics Chromatin 14 (1), 4. 10.1186/s13072-020-00379-z 33407738 PMC7789248

[B70] Herman J. G. Baylin S. B. (2000). Promoter-region hypermethylation and gene silencing in human cancer. Curr. Top. Microbiol. Immunol. 249, 35–54. 10.1007/978-3-642-59696-4_3 10802937

[B71] Herpin A. Schartl M. Depincé A. Guiguen Y. Bobe J. Hua-Van A. (2021). Allelic diversification after transposable element exaptation promoted gsdf as the master sex determining gene of sablefish. Genome Res. 31 (8), 1366–1380. 10.1101/gr.274266.120 34183453 PMC8327909

[B72] Hillestad B. Kristjánsson Ó H. Makvandi-Nejad S. Moghadam H. K. (2020a). Genome-wide association study confirms previous findings of major loci affecting resistance to piscine myocarditis virus in atlantic salmon (*Salmo salar* L). Genes (Basel) 11 (6), 608. 10.3390/genes11060608 PMC7349847 32486315

[B73] Hillestad B. Makvandi-Nejad S. Krasnov A. Moghadam H. K. (2020b). Identification of genetic loci associated with higher resistance to pancreas disease (PD) in Atlantic salmon (*Salmo salar* L). BMC Genomics 21 (1), 388. 10.1186/s12864-020-06788-4 32493246 PMC7268189

[B74] Hillestad B. Moghadam H. K. (2019). Genome-wide association study of piscine myocarditis virus (PMCV) resistance in atlantic salmon (*Salmo salar*). J. Hered. 110 (6), 720–726. 10.1093/jhered/esz040 31287547

[B75] Houston R. D. Bean T. P. Macqueen D. J. Gundappa M. K. Jin Y. H. Jenkins T. L. (2020). Harnessing genomics to fast-track genetic improvement in aquaculture. Nat. Rev. Genet. 21 (7), 389–409. 10.1038/s41576-020-0227-y 32300217

[B76] Houston R. D. Haley C. S. Hamilton A. Guy D. R. Mota-Velasco J. C. Gheyas A. A. (2010). The susceptibility of Atlantic salmon fry to freshwater infectious pancreatic necrosis is largely explained by a major QTL. Heredity 105 (3), 318–327. 10.1038/hdy.2009.171 19935825

[B77] Houston R. D. Haley C. S. Hamilton A. Guy D. R. Tinch A. E. Taggart J. B. (2008). Major quantitative trait loci affect resistance to infectious pancreatic necrosis in Atlantic salmon (*Salmo salar*). Genetics 178 (2), 1109–1115. 10.1534/genetics.107.082974 18245341 PMC2248365

[B78] Huang R. Sun J. Luo Q. He L. Liao L. Li Y. (2015). Genetic variations of body weight and GCRV resistance in a random mating population of grass carp. Oncotarget 6 (34), 35433–35442. 10.18632/oncotarget.5945 26439690 PMC4742116

[B79] Huang Y. Wen H. Zhang M. Hu N. Si Y. Li S. (2018). The DNA methylation status of MyoD and IGF-I genes are correlated with muscle growth during different developmental stages of Japanese flounder (*Paralichthys olivaceus*). Comp. Biochem. Physiol. B Biochem. Mol. Biol. 219-220, 33–43. 10.1016/j.cbpb.2018.02.005 29486246

[B80] Ito S. D'Alessio A. C. Taranova O. V. Hong K. Sowers L. C. Zhang Y. (2010). Role of Tet proteins in 5mC to 5hmC conversion, ES-cell self-renewal and inner cell mass specification. Nature 466 (7310), 1129–1133. 10.1038/nature09303 20639862 PMC3491567

[B81] Ito S. Shen L. Dai Q. Wu S. C. Collins L. B. Swenberg J. A. (2011). Tet proteins can convert 5-methylcytosine to 5-formylcytosine and 5-carboxylcytosine. Science 333 (6047), 1300–1303. 10.1126/science.1210597 21778364 PMC3495246

[B82] Jaafar R. Ødegård J. Mathiessen H. Karami A. M. Marana M. H. von Gersdorff Jørgensen L. (2020). Quantitative trait loci (QTL) associated with resistance of rainbow trout *Oncorhynchus mykiss* against the parasitic ciliate *Ichthyophthirius multifiliis* . J. Fish. Dis. 43 (12), 1591–1602. 10.1111/jfd.13264 32944955 PMC7692903

[B83] Jackson T. R. Ferguson M. M. Danzmann R. G. Fishback A. G. Ihssen P. E. O’Connell M. (1998). Identification of two QTL influencing upper temperature tolerance in three rainbow trout (*Oncorhynchus mykiss*) half-sib families. Hered. (Edinb) 80, 143–151. 10.1046/j.1365-2540.1998.00289.x

[B84] Jeremias G. Barbosa J. Marques S. M. De Schamphelaere K. A. C. Van Nieuwerburgh F. Deforce D. (2018). Transgenerational inheritance of DNA hypomethylation in *Daphnia magna* in response to salinity stress. Environ. Sci. Technol. 52 (17), 10114–10123. 10.1021/acs.est.8b03225 30113818

[B85] Jessop P. Ruzov A. Gering M. (2018). Developmental functions of the dynamic DNA methylome and hydroxymethylome in the mouse and zebrafish: Similarities and differences. Front. Cell Dev. Biol. 6, 27. 10.3389/fcell.2018.00027 29616219 PMC5869911

[B86] Jia Y. Zheng J. Chi M. Liu S. Jiang W. Cheng S. (2019). Molecular identification of dmrt1 and its promoter CpG methylation in correlation with gene expression during gonad development in Culter alburnus. Fish. Physiol. Biochem. 45 (1), 245–252. 10.1007/s10695-018-0558-1 30276577

[B87] Jiang Q. Qian L. Gu S. Guo X. Zhang X. Sun L. (2020). Investigation of growth retardation in *Macrobrachium rosenbergii* based on genetic/epigenetic variation and molt performance. Comp. Biochem. Physiol. Part D. Genomics Proteomics 35, 100683. 10.1016/j.cbd.2020.100683 32279060

[B88] Jin B. Robertson K. D. (2013). DNA methyltransferases, DNA damage repair, and cancer. Adv. Exp. Med. Biol. 754, 3–29. 10.1007/978-1-4419-9967-2_1 22956494 PMC3707278

[B89] Jin S. Bian C. Jiang S. Han K. Xiong Y. Zhang W. (2021). A chromosome-level genome assembly of the oriental river prawn. Macrobrachium Nippon. Gigascience. 10 (1), giaa160. 10.1093/gigascience/giaa160 PMC7812440 33459341

[B90] Jin Y. Zhou T. Geng X. Liu S. Chen A. Yao J. (2017). A genome-wide association study of heat stress-associated SNPs in catfish. Anim. Genet. 48 (2), 233–236. 10.1111/age.12482 27476875

[B91] Jin Y. Zhou T. Jiang W. Li N. Xu X. Tan S. (2022). Allelically and differentially expressed genes after infection of *Edwardsiella ictaluri*in channel catfish as determined by bulk segregant RNA-seq. Mar. Biotechnol. 24 (1), 174–189. 10.1007/s10126-022-10094-3 35166964

[B92] Jones P. A. (2012). Functions of DNA methylation: Islands, start sites, gene bodies and beyond. Nat. Rev. Genet. 13 (7), 484–492. 10.1038/nrg3230 22641018

[B93] Kamiya T. Kai W. Tasumi S. Oka A. Matsunaga T. Mizuno N. (2012). A trans-species missense SNP in Amhr2 is associated with sex determination in the tiger pufferfish, *Takifugu rubripes* (fugu). PLoS Genet. 8 (7), e1002798. 10.1371/journal.pgen.1002798 22807687 PMC3395601

[B94] Kamstra J. H. Sales L. B. Aleström P. Legler J. (2017). Differential DNA methylation at conserved non-genic elements and evidence for transgenerational inheritance following developmental exposure to mono(2-ethylhexyl) phthalate and 5-azacytidine in zebrafish. Epigenetics Chromatin 10, 20. 10.1186/s13072-017-0126-4 28413451 PMC5389146

[B95] Kang Y. Kim Y. W. Kang J. Kim A. (2021). Histone H3K4me1 and H3K27ac play roles in nucleosome eviction and eRNA transcription, respectively, at enhancers. FASEB J. 35 (8), e21781. 10.1096/fj.202100488R 34309923

[B96] Kelley J. L. Tobler M. Beck D. Sadler-Riggleman I. Quackenbush C. R. Arias Rodriguez L. (2021). Epigenetic inheritance of DNA methylation changes in fish living in hydrogen sulfide-rich springs. Proc. Natl. Acad. Sci. U. S. A. 118 (26), e2014929118. 10.1073/pnas.2014929118 34185679 PMC8255783

[B97] Kenchanmane Raju S. K. Ritter E. J. Niederhuth C. E. (2019). Establishment, maintenance, and biological roles of non-CG methylation in plants. Essays Biochem. 63 (6), 743–755. 10.1042/EBC20190032 31652316 PMC6923318

[B98] Knecht A. L. Truong L. Marvel S. W. Reif D. M. Garcia A. Lu C. (2017). Transgenerational inheritance of neurobehavioral and physiological deficits from developmental exposure to benzo[a]pyrene in zebrafish. Toxicol. Appl. Pharmacol. 329, 148–157. 10.1016/j.taap.2017.05.033 28583304 PMC5539966

[B99] Konstantinidis I. Anastasiadi D. Sætrom P. Nedoluzhko A. V. Mjelle R. Podgorniak T. (2021). Epigenetic mapping of the somatotropic axis in Nile tilapia reveals differential DNA hydroxymethylation marks associated with growth. Genomics 113 (5), 2953–2964. 10.1016/j.ygeno.2021.06.037 34214627 PMC7611323

[B100] Koyama T. Nakamoto M. Morishima K. Yamashita R. Yamashita T. Sasaki K. (2019). A SNP in a steroidogenic enzyme is associated with phenotypic sex in Seriola fishes. Curr. Biol. 29 (11), 1901–1909. 10.1016/j.cub.2019.04.069 31130458

[B101] Kratochwil C. F. Meyer A. (2015). Mapping active promoters by ChIP-seq profiling of H3K4me3 in cichlid fish - a first step to uncover cis-regulatory elements in ecological model teleosts. Mol. Ecol. Resour. 15 (4), 761–771. 10.1111/1755-0998.12350 25403420

[B102] Kvamme B. O. Gadan K. Finne-Fridell F. Niklasson L. Sundh H. Sundell K. (2013). Modulation of innate immune responses in Atlantic salmon by chronic hypoxia-induced stress. Fish. Shellfish Immunol. 34 (1), 55–65. 10.1016/j.fsi.2012.10.006 23085636

[B103] Kvist J. Gonçalves Athanàsio C. Shams Solari O. Brown J. B. Colbourne J. K. Pfrender M. E. (2018). Pattern of DNA methylation in Daphnia: Evolutionary perspective. Genome Biol. Evol. 10 (8), 1988–2007. 10.1093/gbe/evy155 30060190 PMC6097596

[B104] Lacal I. Ventura R. (2018). Epigenetic inheritance: Concepts, mechanisms and perspectives. Front. Mol. Neurosci. 11, 292. 10.3389/fnmol.2018.00292 30323739 PMC6172332

[B105] Laing L. V. Viana J. Dempster E. L. Uren Webster T. M. van Aerle R. Mill J. (2018). Sex-specific transcription and DNA methylation profiles of reproductive and epigenetic associated genes in the gonads and livers of breeding zebrafish. Comp. Biochem. Physiol. A Mol. Integr. Physiol. 222, 16–25. 10.1016/j.cbpa.2018.04.004 29655816

[B106] Laufer B. I. Singh S. M. (2015). Strategies for precision modulation of gene expression by epigenome editing: An overview. Epigenetics Chromatin 8, 34. 10.1186/s13072-015-0023-7 26388942 PMC4574080

[B107] Lev Maor G. Yearim A. Ast G. (2015). The alternative role of DNA methylation in splicing regulation. Trends Genet. 31 (5), 274–280. 10.1016/j.tig.2015.03.002 25837375

[B108] Li C. G. Wang H. Chen H. J. Zhao Y. Fu P. S. Ji X. S. (2014). Differential expression analysis of genes involved in high-temperature induced sex differentiation in Nile tilapia. Comp. Biochem. Physiol. B Biochem. Mol. Biol. 177-178, 36–45. 10.1016/j.cbpb.2014.08.006 25199961

[B109] Li H. L. Gu X. H. Li B. J. Chen C. H. Lin H. R. Xia J. H. (2017). Genome-wide QTL analysis identified significant associations between hypoxia tolerance and mutations in the GPR132 and ABCG4 genes in nile Tilapia. Mar. Biotechnol. 19 (5), 441–453. 10.1007/s10126-017-9762-8 28698960

[B110] Li J. Boroevich K. A. Koop B. F. Davidson W. S. (2011). Comparative genomics identifies candidate genes for infectious salmon anemia (ISA) resistance in Atlantic salmon (*Salmo salar*). Mar. Biotechnol. (NY) 13 (2), 232–241. 10.1007/s10126-010-9284-0 20396924 PMC3084937

[B111] Li M. Sun Y. Zhao J. Shi H. Zeng S. Ye K. (2015). A tandem duplicate of anti-müllerian hormone with a missense SNP on the Y chromosome is essential for male sex determination in nile Tilapia, *Oreochromis niloticus* . PLoS Genet. 11 (11), e1005678. 10.1371/journal.pgen.1005678 26588702 PMC4654491

[B112] Li N. Zhou T. Geng X. Jin Y. Wang X. Liu S. (2018). Identification of novel genes significantly affecting growth in catfish through GWAS analysis. Mol. Genet. Genomics 293 (3), 587–599. 10.1007/s00438-017-1406-1 29230585

[B113] Li P. Chen J. Zhu C. Pan Z. Li Q. Wei H. (2022a). DNA methylation difference between female and male ussuri catfish (pseudobagrus ussuriensis) in brain and gonad tissues. Life (Basel) 12 (6), 874. 10.3390/life12060874 35743904 PMC9228513

[B114] Li X. Liu B. Yang J. Li G. Wen H. Zhang M. (2022b). DNA methylation in promoter region of immune related genes STAT3 and VEGFA and biochemical parameters change in muscle of Japanese flounder under acute hypoxia. Dev. Comp. Immunol. 129, 104295. 10.1016/j.dci.2021.104295 34662685

[B115] Li Y. Zhang B. Yang Y. Chen S. (2019). Estimationof genetic parameters for juvenile growth performance traits in oliveflounder (*Paralichthys olivaceus*). Aquac. Fish. 4 (2), 48–52. 10.1016/j.aaf.2018.12.001

[B116] Lien S. Koop B. F. Sandve S. R. Miller J. R. Kent M. P. Nome T. (2016). The Atlantic salmon genome provides insights into rediploidization. Nature 533 (7602), 200–205. 10.1038/nature17164 27088604 PMC8127823

[B117] Lighten J. Incarnato D. Ward B. J. van Oosterhout C. Bradbury I. Hanson M. (2016). Adaptive phenotypic response to climate enabled by epigenetics in a K-strategy species, the fish *Leucoraja ocellata* (Rajidae). R. Soc. Open Sci. 3 (10), 160299. 10.1098/rsos.160299 27853546 PMC5098971

[B118] Liu F. Sun F. Xia J. H. Li J. Fu G. H. Lin G. (2014). A genome scan revealed significant associations of growth traits with a major QTL and GHR2 in tilapia. Sci. Rep. 4, 7256. 10.1038/srep07256 25435025 PMC4248272

[B119] Liu H. Chen C. Lv M. Liu N. Hu Y. Zhang H. (2021). A chromosome-level assembly of blunt snout bream (*Megalobrama amblycephala*) genome reveals an expansion of olfactory receptor genes in freshwater fish. Mol. Biol. Evol. 38 (10), 4238–4251. 10.1093/molbev/msab152 34003267 PMC8476165

[B120] Liu P. Wang L. Wong S. M. Yue G. H. (2016). Fine mapping QTL for resistance to VNN disease using a high-density linkage map in Asian seabass. Sci. Rep. 6, 32122. 10.1038/srep32122 27555039 PMC4995370

[B121] Liu S. Martin K. E. Gao G. Long R. Evenhuis J. P. Leeds T. D. (2022). Identification of haplotypes associated with resistance to bacterial cold water disease in rainbow trout using whole-genome resequencing. Front. Genet. 13, 936806. 10.3389/fgene.2022.936806 35812729 PMC9260151

[B122] Liu S. Vallejo R. L. Palti Y. Gao G. Marancik D. P. Hernandez A. G. (2015). Identification of single nucleotide polymorphism markers associated with bacterial cold water disease resistance and spleen size in rainbow trout. Front. Genet. 6, 298. 10.3389/fgene.2015.00298 26442114 PMC4585308

[B123] Liu S. Wang X. Sun F. Zhang J. Feng J. Liu H. (2013). RNA-Seq reveals expression signatures of genes involved in oxygen transport, protein synthesis, folding, and degradation in response to heat stress in catfish. Physiol. Genomics 45 (12), 462–476. 10.1152/physiolgenomics.00026.2013 23632418

[B124] Liu Y. Wang W. Liang S. Wang L. Zou Y. Wu Z. (2021). Sexual dimorphism of DNA and histone methylation profiles in the gonads of the olive flounder *Paralichthys olivaceus* . Fish. Physiol. Biochem. 47 (5), 1341–1352. 10.1007/s10695-021-00986-x 34264445

[B125] Liu Z. Liu S. Yao J. Bao L. Zhang J. Li Y. (2016). The channel catfish genome sequence provides insights into the evolution of scale formation in teleosts. Nat. Commun. 7, 11757. 10.1038/ncomms11757 27249958 PMC4895719

[B126] Loughland I. Little A. Seebacher F. (2021). DNA methyltransferase 3a mediates developmental thermal plasticity. BMC Biol. 19 (1), 11. 10.1186/s12915-020-00942-w 33478487 PMC7819298

[B127] Luo C. Hajkova P. Ecker J. R. (2018). Dynamic DNA methylation: In the right place at the right time. Science 361 (6409), 1336–1340. 10.1126/science.aat6806 30262495 PMC6197482

[B128] Ma A. Huang Z. Wang X. A. Xu Y. Guo X. (2021). Identification of quantitative trait loci associated with upper temperature tolerance in turbot, *Scophthalmus maximus* . Sci. Rep. 11 (1), 21920. 10.1038/s41598-021-01062-3 34753974 PMC8578632

[B129] Ma X. Su B. Bangs M. Alston V. Backenstose N. J. C. Simora R. M. (2021). Comparative genomic and transcriptomic analyses revealed twenty-six candidate genes involved in the air-breathing development and function of the bighead catfish *Clarias macrocephalus* . Mar. Biotechnol. 23 (1), 90–105. 10.1007/s10126-020-10005-4 33113010

[B130] Marchione A. D. Thompson Z. Kathrein K. L. (2021). DNA methylation and histone modifications are essential for regulation of stem cell formation and differentiation in zebrafish development. Brief. Funct. Genomics, elab022. 10.1093/bfgp/elab022 33782688

[B131] Massault C. Franch R. Haley C. de Koning D. J. Bovenhuis H. Pellizzari C. (2011). Quantitative trait loci for resistance to fish pasteurellosis in gilthead sea bream (*Sparus aurata*). Anim. Genet. 42 (2), 191–203. 10.1111/j.1365-2052.2010.02110.x 20946317

[B132] Matos I. M. N. Coelho M. M. Schartl M. (2016). Gene copy silencing and DNA methylation in natural and artificially produced allopolyploid fish. J. Exp. Biol. 219 (19), 3072–3081. 10.1242/jeb.140418 27445349

[B133] Matsuda M. Nagahama Y. Shinomiya A. Sato T. Matsuda C. Kobayashi T. (2002). DMY is a Y-specific DM-domain gene required for male development in the medaka fish. Nature 417 (6888), 559–563. 10.1038/nature751 12037570

[B134] Matsuda M. Nagahama Y. Kobayashi T. Matsuda C. Hamaguchi S. Sakaizumi M. (2003). The sex determining gene of medaka: A Y-specific DM domain gene (DMY) is required for male development. Fish Physiology Biochem. 28, 135–139. 10.1023/B:FISH.0000030500.29914.7a

[B135] Medina-Gali R. Belló-Pérez M. Martínez-López A. Falcó A. Ortega-Villaizan M. M. Encinar J. A. (2018). Chromatin immunoprecipitation and high throughput sequencing of SVCV-infected zebrafish reveals novel epigenetic histone methylation patterns involved in antiviral immune response. Fish. Shellfish Immunol. 82, 514–521. 10.1016/j.fsi.2018.08.056 30170110

[B136] Melamed-Bessudo C. Levy A. A. (2012). Deficiency in DNA methylation increases meiotic crossover rates in euchromatic but not in heterochromatic regions in Arabidopsis. Proc. Natl. Acad. Sci. U. S. A. 109 (16), E981–E988. 10.1073/pnas.1120742109 22460791 PMC3341010

[B137] Metzger D. C. Schulte P. M. (2016). Epigenomics in marine fishes. Mar. Genomics 30, 43–54. 10.1016/j.margen.2016.01.004 26833273

[B138] Metzger D. C. H. Schulte P. M. (2018). The DNA methylation landscape of stickleback reveals patterns of sex chromosome evolution and effects of environmental salinity. Genome Biol. Evol. 10 (3), 775–785. 10.1093/gbe/evy034 29420714 PMC5841383

[B139] Moen T. Baranski M. Sonesson A. K. Kjøglum S. (2009). Confirmation and fine-mapping of a major QTL for resistance to infectious pancreatic necrosis in Atlantic salmon (*Salmo salar*): Population-level associations between markers and trait. BMC Genomics 10, 368. 10.1186/1471-2164-10-368 19664221 PMC2728743

[B140] Moen T. Sonesson A. K. Hayes B. Lien S. Munck H. Meuwissen T. H. (2007). Mapping of a quantitative trait locus for resistance against infectious salmon anaemia in atlantic salmon (*Salmo salar*): Comparing survival analysis with analysis on affected/resistant data. BMC Genet. 8, 53. 10.1186/1471-2156-8-53 17697344 PMC2000910

[B141] Moen T. Torgersen J. Santi N. Davidson W. S. Baranski M. Ødegård J. (2015). Epithelial cadherin determines resistance to infectious pancreatic necrosis virus in atlantic salmon. Genetics 200 (4), 1313–1326. 10.1534/genetics.115.175406 26041276 PMC4574245

[B142] Moore L. D. Le T. Fan G. (2013). DNA methylation and its basic function. Neuropsychopharmacology 38 (1), 23–38. 10.1038/npp.2012.112 22781841 PMC3521964

[B143] Myosho T. Otake H. Masuyama H. Matsuda M. Kuroki Y. Fujiyama A. (2012). Tracing the emergence of a novel sex-determining gene in medaka, *Oryzias luzonensis* . Genetics 191 (1), 163–170. 10.1534/genetics.111.137497 22367037 PMC3338257

[B144] Mzula A. Wambura P. N. Mdegela R. H. Shirima G. M. (2021). Present status of aquaculture and the challenge of bacterial diseases in freshwater farmed fish in Tanzania; A call for sustainable strategies. Aquac. Fish. 6 (3), 247–253. 10.1016/j.aaf.2020.05.003

[B145] Nakamura M. Gao Y. Dominguez A. A. Qi L. S. (2021). CRISPR technologies for precise epigenome editing. Nat. Cell Biol. 23 (1), 11–22. 10.1038/s41556-020-00620-7 33420494

[B146] Nanda I. Kondo M. Hornung U. Asakawa S. Winkler C. Shimizu A. (2002). A duplicated copy of DMRT1 in the sex-determining region of the Y chromosome of the medaka, *Oryzias latipes* . Proc. Natl. Acad. Sci. U. S. A. 99 (18), 11778–11783. 10.1073/pnas.182314699 12193652 PMC129345

[B147] Navarro-Martín L. Viñas J. Ribas L. Díaz N. Gutiérrez A. Di Croce L. (2011). DNA methylation of the gonadal aromatase (cyp19a) promoter is involved in temperature-dependent sex ratio shifts in the European sea bass. PLoS Genet. 7 (12), e1002447. 10.1371/journal.pgen.1002447 22242011 PMC3248465

[B148] Naylor R. L. Hardy R. W. Buschmann A. H. Bush S. R. Cao L. Klinger D. H. (2021). A 20-year retrospective review of global aquaculture. Nature 591 (7851), 551–563. 10.1038/s41586-021-03308-6 33762770

[B149] Norouzitallab P. Baruah K. Vandegehuchte M. Van Stappen G. Catania F. Vanden Bussche J. (2014). Environmental heat stress induces epigenetic transgenerational inheritance of robustness in parthenogenetic Artemia model. FASEB J. 28 (8), 3552–3563. 10.1096/fj.14-252049 24755740

[B150] Nozawa K. Lin Y. Kubodera R. Shimizu Y. Tanaka H. Ohshima T. (2017). Zebrafish Mecp2 is required for proper axonal elongation of motor neurons and synapse formation. Dev. Neurobiol. 77 (9), 1101–1113. 10.1002/dneu.22498 28371371

[B151] Ooga M. Inoue A. Kageyama S. Akiyama T. Nagata M. Aoki F. (2008). Changes in H3K79 methylation during preimplantation development in mice. Biol. Reprod. 78 (3), 413–424. 10.1095/biolreprod.107.063453 18003948

[B152] Oqani R. K. Lin T. Lee J. E. Kang J. W. Shin H. Y. (2019). Il Jin D. Iws1 and Spt6 regulate trimethylation of histone H3 on lysine 36 through akt signaling and are essential for mouse embryonic genome activation. Sci. Rep. 9 (1), 3831. 10.1038/s41598-019-40358-3 30846735 PMC6405902

[B153] Osborn T. C. Pires J. C. Birchler J. A. Auger D. L. Chen Z. J. Lee H. S. (2003). Understanding mechanisms of novel gene expression in polyploids. Trends Genet. 19 (3), 141–147. 10.1016/s0168-9525(03)00015-5 12615008

[B154] Ou M. Mao H. Luo Q. Zhao J. Liu H. Zhu X. (2019). The DNA methylation level is associated with the superior growth of the hybrid fry in snakehead fish (*Channa argus* × *Channa maculata*). Gene 703, 125–133. 10.1016/j.gene.2019.03.072 30978477

[B155] Palaiokostas C. Robledo D. Vesely T. Prchal M. Pokorova D. Piackova V. (2018). Mapping and sequencing of a significant quantitative trait locus affecting resistance to koi herpesvirus in common carp. G3 (Bethesda) 8 (11), 3507–3513. 10.1534/g3.118.200593 30150301 PMC6222565

[B156] Palti Y. Vallejo R. L. Gao G. Liu S. Hernandez A. G. Rexroad C. E. III (2015). Detection and validation of QTL affecting bacterial cold water disease resistance in rainbow trout using restriction-site associated DNA sequencing. PloS one 10 (9), e0138435. 10.1371/journal.pone.0138435 26376182 PMC4574402

[B157] Pan Q. Feron R. Yano A. Guyomard R. Jouanno E. Vigouroux E. (2019). Identification of the master sex determining gene in Northern pike (*Esox lucius*) reveals restricted sex chromosome differentiation. PLoS Genet. 15 (8), e1008013. 10.1371/journal.pgen.1008013 31437150 PMC6726246

[B158] Pavelin J. Jin Y. H. Gratacap R. L. Taggart J. B. Hamilton A. Verner-Jeffreys D. W. (2021). The nedd-8 activating enzyme gene underlies genetic resistance to infectious pancreatic necrosis virus in Atlantic salmon. Genomics 113 (6), 3842–3850. 10.1016/j.ygeno.2021.09.012 34547402 PMC8682971

[B159] Perry G. M. Danzmann R. G. Ferguson M. M. Gibson J. P. (2001). Quantitative trait loci for upper thermal tolerance in outbred strains of rainbow trout (*Oncorhynchus mykiss*). Hered. (Edinb) 86 (3), 333–341. 10.1046/j.1365-2540.2001.00838.x 11488970

[B160] Pierron F. Lorioux S. Héroin D. Daffe G. Etcheverria B. Cachot J. (2021). Transgenerational epigenetic sex determination: Environment experienced by female fish affects offspring sex ratio. Environ. Pollut. 277, 116864. 10.1016/j.envpol.2021.116864 33714788

[B161] Podgorniak T. Brockmann S. Konstantinidis I. Fernandes J. M. O. (2019). Differences in the fast muscle methylome provide insight into sex-specific epigenetic regulation of growth in Nile tilapia during early stages of domestication. Epigenetics 14 (8), 818–836. 10.1080/15592294.2019.1618164 31131688 PMC6597363

[B162] Potok Magdalena E. Nix David A. Parnell Timothy J. Cairns Bradley R. (2013). Reprogramming the maternal zebrafish genome after fertilization to match the paternal methylation pattern. Cell 153, 759–772. 10.1016/j.cell.2013.04.030 23663776 PMC4030421

[B163] Qian X. Ba Y. Zhuang Q. Zhong G. (2014). RNA-Seq technology and its application in fish transcriptomics. OMICS 18 (2), 98–110. 10.1089/omi.2013.0110 24380445 PMC3920896

[B164] Quinn N. L. McGowan C. R. Cooper G. A. Koop B. F. Davidson W. S. (2011). Identification of genes associated with heat tolerance in Arctic charr exposed to acute thermal stress. Physiol. Genomics 43 (11), 685–696. 10.1152/physiolgenomics.00008.2011 21467159

[B165] Ran Z. Li Z. Yan X. Liao K. Kong F. Zhang L. (2019). Chromosome-level genome assembly of the razor clam *Sinonovacula constricta* (Lamarck, 1818). Mol. Ecol. Resour. 19, 1647–1658. 10.1111/1755-0998.13086 31483923

[B166] Ren L. Zhang H. Luo M. Gao X. Cui J. Zhang X. (2022). Heterosis of growth trait regulated by DNA methylation and miRNA in allotriploid fish. Epigenetics Chromatin 15 (1), 19. 10.1186/s13072-022-00455-6 35597966 PMC9123727

[B167] Rexroad C. Vallet J. Matukumalli L. K. Reecy J. Bickhart D. Blackburn H. (2019). Genome to phenome: Improving animal health, production, and well-being - a new USDA blueprint for animal genome research 2018-2027. Front. Genet. 10, 327. 10.3389/fgene.2019.00327 31156693 PMC6532451

[B168] Ribas L. Vanezis K. Imués M. A. Piferrer F. (2017). Treatment with a DNA methyltransferase inhibitor feminizes zebrafish and induces long-term expression changes in the gonads. Epigenetics Chromatin 10 (1), 59. 10.1186/s13072-017-0168-7 29216900 PMC5721477

[B169] Robinson N. A. Johnsen H. Moghadam H. Andersen Ø. Tveiten H. (2019). Early developmental stress affects subsequent gene expression response to an acute stress in atlantic salmon: An approach for creating robust fish for aquaculture? G3 (Bethesda) 9 (5), 1597–1611. 10.1534/g3.119.400152 30885921 PMC6505151

[B170] Robledo D. Gutiérrez A. P. Barría A. Lhorente J. P. Houston R. D. Yáñez J. M. (2019). Discovery and functional annotation of quantitative trait loci affecting resistance to sea lice in atlantic salmon. Front. Genet. 10, 56. 10.3389/fgene.2019.00056 30800143 PMC6375901

[B171] Rodríguez-Ramilo S. T. Toro M. A. Bouza C. Hermida M. Pardo B. G. Cabaleiro S. (2011). QTL detection for *Aeromonas salmonicida* resistance related traits in turbot (*Scophthalmus maximus*). BMC Genomics 12, 541. 10.1186/1471-2164-12-541 22047500 PMC3216323

[B172] Roy S. Baruah K. Bossier P. Vanrompay D. Norouzitallab P. (2022). Induction of transgenerational innate immune memory against *Vibrio* infections in a brine shrimp (*Artemia franciscana*) model. Aquaculture 557, 738309. 10.1016/j.aquaculture.2022.738309

[B173] Roy S. Kumar V. Behera B. K. Das B. K. (2021). “Epigenetics: Perspectives and potential in aquaculture,” in Advances in Fisheries Biotechnology. 2021. Editors Pandey P. K. Parhi J. (Singapore: Springer). 10.1007/978-981-16-3215-0_9

[B174] Roy S. Kumar V. Bossier P. Norouzitallab P. Vanrompay D. (2019). Phloroglucinol treatment induces transgenerational epigenetic inherited resistance against *Vibrio* infections and thermal stress in a brine shrimp (*Artemia franciscana*) model. Front. Immunol. 10, 2745. 10.3389/fimmu.2019.02745 31827471 PMC6890837

[B175] Sato Y. Kujirai T. Arai R. Asakawa H. Ohtsuki C. Horikoshi N. (2016). A genetically encoded probe for live-cell imaging of H4K20 monomethylation. J. Mol. Biol. 428 (20), 3885–3902. 10.1016/j.jmb.2016.08.010 27534817

[B176] Shang X. Wan Q. Su J. Su J. (2016). DNA methylation of CiRIG-I gene notably relates to the resistance against GCRV and negatively-regulates mRNA expression in grass carp, *Ctenopharyngodon idella* . Immunobiology 221 (1), 23–30. 10.1016/j.imbio.2015.08.006 26314762

[B177] Shao C. Bao B. Xie Z. Chen X. Li B. Jia X. (2017). The genome and transcriptome of Japanese flounder provide insights into flatfish asymmetry. Nat. Genet. 49 (1), 119–124. 10.1038/ng.3732 27918537

[B178] Shao C. Li Q. Chen S. Zhang P. Lian J. Hu Q. (2014). Epigenetic modification and inheritance in sexual reversal of fish. Genome Res. 24 (4), 604–615. 10.1101/gr.162172.113 24487721 PMC3975060

[B179] Shi H. Zhou T. Wang X. Yang Y. Wu C. Liu S. (2018). Genome-wide association analysis of intra-specific QTL associated with the resistance for enteric septicemia of catfish. Mol. Genet. Genomics 293 (6), 1365–1378. 10.1007/s00438-018-1463-0 29967962

[B180] Simon J. M. Parker J. S. Liu F. Rothbart S. B. Ait-Si-Ali S. Strahl B. D. (2015). A role for widely interspaced zinc finger (WIZ) in retention of the G9a methyltransferase on chromatin. J. Biol. Chem. 290 (43), 26088–26102. 10.1074/jbc.M115.654459 26338712 PMC4646261

[B181] Skinner M. K. Guerrero-Bosagna C. (2009). Environmental signals and transgenerational epigenetics. Epigenomics 1 (1), 111–117. 10.2217/epi.09.11 20563319 PMC2886501

[B182] Skvortsova K. Iovino N. Bogdanović O. (2018). Functions and mechanisms of epigenetic inheritance in animals. Nat. Rev. Mol. Cell Biol. 19 (12), 774–790. 10.1038/s41580-018-0074-2 30425324

[B183] Somorjai I. M. Danzmann R. G. Ferguson M. M. (2003). Distribution of temperature tolerance quantitative trait loci in Arctic charr (*Salvelinus alpinus*) and inferred homologies in rainbow trout (*Oncorhynchus mykiss*). Genetics 165 (3), 1443–1456. 10.1093/genetics/165.3.1443 14668393 PMC1462839

[B184] Song W. Xie Y. Sun M. Li X. Fitzpatrick C. K. Vaux F. (2021). A duplicated amh is the master sex-determining gene for Sebastes rockfish in the Northwest Pacific. Open Biol. 11 (7), 210063. 10.1098/rsob.210063 34255977 PMC8277470

[B185] Star B. Nederbragt A. J. Jentoft S. Grimholt U. Malmstrøm M. Gregers T. F. (2011). The genome sequence of Atlantic cod reveals a unique immune system. Nature 477 (7363), 207–210. 10.1038/nature10342 21832995 PMC3537168

[B186] Sun D. Yu H. Li Q. (2022). Genome-wide differential DNA methylomes provide insights into the infertility of triploid oysters. Mar. Biotechnol. 24 (1), 18–31. 10.1007/s10126-021-10083-y 35041105

[B187] Sun L. Liu S. Bao L. Li Y. Feng J. Liu Z. (2015). Claudin multigene family in channel catfish and their expression profiles in response to bacterial infection and hypoxia as revealed by meta-analysis of RNA-Seq datasets. Comp. Biochem. Physiol. Part D. Genomics Proteomics 13, 60–69. 10.1016/j.cbd.2015.01.002 25681604

[B188] Sun L. Liu S. Wang R. Jiang Y. Zhang Y. Zhang J. (2014). Identification and analysis of genome-wide SNPs provide insight into signatures of selection and domestication in channel catfish (*Ictalurus punctatus*). PloS one 9 (10), e109666. 10.1371/journal.pone.0109666 25313648 PMC4196944

[B189] Sun L. X. Wang Y. Y. Zhao Y. Wang H. Li N. Ji X. S. (2016). Global DNA methylation changes in nile Tilapia gonads during high temperature-induced masculinization. PLoS One 11 (8), e0158483. 10.1371/journal.pone.0158483 27486872 PMC4972363

[B190] Tadmor-Levi R. Doron-Faigenboim A. Marcos-Hadad E. Petit J. Hulata G. Forlenza M. (2019a). Different transcriptional response between susceptible and resistant common carp (*Cyprinus carpio*) fish hints on the mechanism of CyHV-3 disease resistance. BMC Genomics 20 (1), 1019. 10.1186/s12864-019-6391-9 31878870 PMC6933926

[B191] Tadmor-Levi R. Hulata G. David L. (2019b). Multiple interacting QTLs affect disease challenge survival in common carp (*Cyprinus carpio*). Hered. (Edinb) 123 (5), 565–578. 10.1038/s41437-019-0224-0 PMC6972736 31036952

[B192] Tahiliani M. Koh K. P. Shen Y. Pastor W. A. Bandukwala H. Brudno Y. (2009). Conversion of 5-methylcytosine to 5-hydroxymethylcytosine in mammalian DNA by MLL partner TET1. Science 324 (5929), 930–935. 10.1126/science.1170116 19372391 PMC2715015

[B193] Takayama K. Shimoda N. Takanaga S. Hozumi S. Kikuchi Y. (2014). Expression patterns of dnmt3aa, dnmt3ab, and dnmt4 during development and fin regeneration in zebrafish. Gene Expr. Patterns 14 (2), 105–110. 10.1016/j.gep.2014.01.005 24509247

[B194] Tan E. Kinoshita S. Suzuki Y. Ineno T. Tamaki K. Kera A. (2016). Different gene expression profiles between normal and thermally selected strains of rainbow trout, *Oncorhynchus mykiss*, as revealed by comprehensive transcriptome analysis. Gene 576 (1), 637–643. 10.1016/j.gene.2015.10.028 26476292

[B195] Tan S. Wang W. Zhong X. Tian C. Niu D. Bao L. (2018a). Increased alternative splicing as a host response to *Edwardsiella ictaluri*infection in catfish. Mar. Biotechnol. 20 (6), 729–738. 10.1007/s10126-018-9844-2 30014301

[B196] Tan S. Zhou T. Wang W. Jin Y. Wang X. Geng X. (2018b). GWAS analysis using interspecific backcross progenies reveals superior blue catfish alleles responsible for strong resistance against enteric septicemia of catfish. Mol. Genet. Genomics 293 (5), 1107–1120. 10.1007/s00438-018-1443-4 29737402

[B197] Tao W. Cao J. Xiao H. Zhu X. Dong J. Kocher T. D. (2021). A chromosome-level genome assembly of Mozambique Tilapia (*Oreochromis mossambicus*) reveals the structure of sex determining regions. Front. Genet. 12, 796211. 10.3389/fgene.2021.796211 34956335 PMC8692795

[B198] Termolino P. Cremona G. Consiglio M. F. Conicella C. (2016). Insights into epigenetic landscape of recombination-free regions. Chromosoma 125 (2), 301–308. 10.1007/s00412-016-0574-9 26801812 PMC4830869

[B199] Thayil A. J. Wang X. Bhandari P. Vom Saal F. S. Tillitt D. E. Bhandari R. K. (2020). Bisphenol A and 17α-ethinylestradiol-induced transgenerational gene expression differences in the brain-pituitary-testis axis of medaka, *Oryzias latipes* . Biol. Reprod. 103 (6), 1324–1335. 10.1093/biolre/ioaa169 32940650 PMC7711903

[B200] Trijau M. Asselman J. Armant O. Adam-Guillermin C. De Schamphelaere K. A. C. Alonzo F. (2018). Transgenerational DNA methylation changes in Daphnia magna exposed to chronic γ irradiation. Environ. Sci. Technol. 52 (7), 4331–4339. 10.1021/acs.est.7b05695 29486114

[B201] Uengwetwanit T. Pootakham W. Nookaew I. Sonthirod C. Angthong P. Sittikankaew K. (2021). A chromosome-level assembly of the black tiger shrimp (*Penaeus monodon*) genome facilitates the identification of growth-associated genes. Mol. Ecol. Resour. 21 (5), 1620–1640. 10.1111/1755-0998.13357 33586292 PMC8197738

[B202] Valdivieso A. Ribas L. Monleón-Getino A. Orbán L. Piferrer F. (2020). Exposure of zebrafish to elevated temperature induces sex ratio shifts and alterations in the testicular epigenome of unexposed offspring. Environ. Res. 186, 109601. 10.1016/j.envres.2020.109601 32371278

[B203] Vallejo R. L. Palti Y. Liu S. Marancik D. P. Wiens G. D. (2014). Validation of linked QTL for bacterial cold water disease resistance and spleen size on rainbow trout chromosome Omy19. Aquaculture 432, 139–143. 10.1016/j.aquaculture.2014.05.003

[B204] Vandegehuchte M. B. De Coninck D. Vandenbrouck T. De Coen W. M. Janssen C. R. (2010a). Gene transcription profiles, global DNA methylation and potential transgenerational epigenetic effects related to Zn exposure history in Daphnia magna. Environ. Pollut. 158 (10), 3323–3329. 10.1016/j.envpol.2010.07.023 20719420

[B205] Vandegehuchte M. B. Lemière F. Janssen C. R. (2009). Quantitative DNA-methylation in *Daphnia magna* and effects of multigeneration Zn exposure. Comp. Biochem. Physiol. C. Toxicol. Pharmacol. 150 (3), 343–348. 10.1016/j.cbpc.2009.05.014 19486948

[B206] Vandegehuchte M. B. Lemière F. Vanhaecke L. Vanden Berghe W. Janssen C. R. (2010b). Direct and transgenerational impact on *Daphnia magna* of chemicals with a known effect on DNA methylation. Comp. Biochem. Physiol. C. Toxicol. Pharmacol. 151 (3), 278–285. 10.1016/j.cbpc.2009.11.007 19961956

[B207] Venney C. J. Wellband K. W. Heath D. D. (2021). Rearing environment affects the genetic architecture and plasticity of DNA methylation in Chinook salmon. Heredity 126 (1), 38–49. 10.1038/s41437-020-0346-4 32699390 PMC7852867

[B208] Veron V. Marandel L. Liu J. Vélez E. J. Lepais O. Panserat S. (2018). DNA methylation of the promoter region of bnip3 and bnip3l genes induced by metabolic programming. BMC Genomics 19 (1), 677. 10.1186/s12864-018-5048-4 30223788 PMC6142374

[B209] Verrier E. R. Dorson M. Mauger S. Torhy C. Ciobotaru C. Hervet C. (2013). Resistance to a rhabdovirus (VHSV) in rainbow trout: Identification of a major QTL related to innate mechanisms. PLoS One 8 (2), e55302. 10.1371/journal.pone.0055302 23390526 PMC3563530

[B210] Vidaurre V. Chen X. (2021). Epigenetic regulation of drosophila germline stem cell maintenance and differentiation. Dev. Biol. 473, 105–118. 10.1016/j.ydbio.2021.02.003 33610541 PMC7992187

[B211] Vogt G. (2022). Studying phenotypic variation and DNA methylation across development, ecology and evolution in the clonal marbled crayfish: A paradigm for investigating epigenotype-phenotype relationships in macro-invertebrates. Sci. Nat. 109 (1), 16. 10.1007/s00114-021-01782-6 35099618

[B212] Waghmare S. G. Samarin A. M. Samarin A. M. Danielsen M. Møller H. S. Policar T. (2021). Histone acetylation dynamics during *in vivo* and *in vitro* oocyte aging in common carp *Cyprinus carpio* . Int. J. Mol. Sci. 22 (11), 6036. 10.3390/ijms22116036 34204879 PMC8199789

[B213] Wan Z. Y. Xia J. H. Lin G. Wang L. Lin V. C. Yue G. H. (2016). Genome-wide methylation analysis identified sexually dimorphic methylated regions in hybrid tilapia. Sci. Rep. 6, 35903. 10.1038/srep35903 27782217 PMC5080608

[B214] Wang E. T. Sandberg R. Luo S. Khrebtukova I. Zhang L. Mayr C. (2008). Alternative isoform regulation in human tissue transcriptomes. Nature 456 (7221), 470–476. 10.1038/nature07509 18978772 PMC2593745

[B215] Wang H. Cui J. Qiu X. Wang X. (2021). Differences in DNA methylation between slow and fast muscle in *Takifugu rubripes* . Gene 801, 145853. 10.1016/j.gene.2021.145853 34274464

[B216] Wang L. Chua E. Sun F. Wan Z. Y. Ye B. Pang H. (2019). Mapping and validating QTL for fatty acid compositions and growth traits in Asian seabass. Mar. Biotechnol. 21 (5), 643–654. 10.1007/s10126-019-09909-7 31273567

[B217] Wang L. Sun F. Wan Z. Y. Yang Z. Tay Y. X. Lee M. (2022). Transposon-induced epigenetic silencing in the X chromosome as a novel form of dmrt1 expression regulation during sex determination in the fighting fish. BMC Biol. 20 (1), 5. 10.1186/s12915-021-01205-y 34996452 PMC8742447

[B218] Wang Q. Hao X. Liu K. Feng B. Li S. Zhang Z. (2020). Early response to heat stress in Chinese tongue sole (*Cynoglossus semilaevis*): Performance of different sexes, candidate genes and networks. BMC Genomics 21 (1), 745. 10.1186/s12864-020-07157-x 33109079 PMC7590793

[B219] Wang Q. Ren X. Liu P. Li J. Lv J. Wang J. (2022). Improved genome assembly of Chinese shrimp (*Fenneropenaeus chinensis*) suggests adaptation to the environment during evolution and domestication. Mol. Ecol. Resour. 22 (1), 334–344. 10.1111/1755-0998.13463 34240531

[B220] Wang S. Zhang J. Jiao W. Li J. Xun X. Sun Y. (2017a). Scallop genome provides insights into evolution of bilaterian karyotype and development. Nat. Ecol. Evol. 1 (5), 120. 10.1038/s41559-017-0120 28812685 PMC10970998

[B221] Wang W. Tan S. Luo J. Shi H. Zhou T. Yang Y. (2019). GWAS analysis indicated importance of NF-κB signaling pathway in host resistance against motile *Aeromonas septicemia* disease in catfish. Mar. Biotechnol. 21 (3), 335–347. 10.1007/s10126-019-09883-0 30895402

[B222] Wang W. Tan S. Yang Y. Zhou T. Xing D. Su B. (2022). Feminization of channel catfish with 17β-oestradiol involves methylation and expression of a specific set of genes independent of the sex determination region. Epigenetics, 1–18. 10.1080/15592294.2022.2086725 PMC9621036 35703353

[B223] Wang X. Liu S. Jiang C. Geng X. Zhou T. Li N. (2017c). Multiple across-strain and within-strain QTLs suggest highly complex genetic architecture for hypoxia tolerance in channel catfish. Mol. Genet. Genomics 292 (1), 63–76. 10.1007/s00438-016-1256-2 27734158

[B224] Wang X. Ma X. Wei G. Ma W. Zhang Z. Chen X. (2021). The Role of DNA methylation reprogramming during sex determination and transition in zebrafish. Genomics Proteomics Bioinforma. 19 (1), 48–63. 10.1016/j.gpb.2020.10.004 PMC8640932 33610791

[B225] Wang Y. Y. Sun L. X. Zhu J. J. Zhao Y. Wang H. Liu H. J. (2017b). Epigenetic control of cyp19a1a expression is critical for high temperature induced Nile tilapia masculinization. J. Therm. Biol. 69, 76–84. 10.1016/j.jtherbio.2017.06.006 29037408

[B226] Wellband K. Roth D. Linnansaari T. Curry R. A. Bernatchez L. (2021). Environment-driven reprogramming of gamete DNA methylation occurs during maturation and is transmitted intergenerationally in Atlantic salmon. G3 (Bethesda) 11 (12), jkab353. 10.1093/g3journal/jkab353 34849830 PMC8664423

[B227] Wen A. Y. You F. Sun P. Li J. Xu D. D. Wu Z. H. (2014). CpG methylation of dmrt1 and cyp19a promoters in relation to their sexual dimorphic expression in the Japanese flounder *Paralichthys olivaceus* . J. Fish. Biol. 84 (1), 193–205. 10.1111/jfb.12277 24372528

[B228] Wiens G. D. Vallejo R. L. Leeds T. D. Palti Y. Hadidi S. Liu S. (2013). Assessment of genetic correlation between bacterial cold water disease resistance and spleen index in a domesticated population of rainbow trout: Identification of QTL on chromosome Omy19. PLoS One 8 (10), e75749. 10.1371/journal.pone.0075749 24130739 PMC3794016

[B229] Wijenayake S. Storey K. B. (2016). The role of DNA methylation during anoxia tolerance in a freshwater turtle (*Trachemys scripta elegans*). J. Comp. Physiol. B 186 (3), 333–342. 10.1007/s00360-016-0960-x 26843075

[B230] Wood R. K. Crowley E. Martyniuk C. J. (2016). Developmental profiles and expression of the DNA methyltransferase genes in the fathead minnow (*Pimephales promelas*) following exposure to di-2-ethylhexyl phthalate. Fish. Physiol. Biochem. 42 (1), 7–18. 10.1007/s10695-015-0112-3 26251286

[B231] Xie J. Sun Y. Cao Y. Han L. Li Y. Ding B. (2022). Transcriptomic and metabolomic analyses provide insights into the growth and development advantages of triploid Apostichopus japonicus. Mar. Biotechnol. 24 (1), 151–162. 10.1007/s10126-022-10093-4 PMC8940865 35122573

[B232] Xu L. Zhao M. Ryu J. H. Hayman E. S. Fairgrieve W. T. Zohar Y. (2022). Reproductive sterility in aquaculture: A review of induction methods and an emerging approach with application to pacific northwest finfish species. Rev. Aquac., 1–22. 10.1111/raq.12712

[B233] Xu P. Zhang X. Wang X. Li J. Liu G. Kuang Y. (2014). Genome sequence and genetic diversity of the common carp, *Cyprinus carpio* . Nat. Genet. 46 (11), 1212–1219. 10.1038/ng.3098 25240282

[B234] Xu Y. Lin H. Yan W. Li J. Sun M. Chen J. (2022). Full-length transcriptome of red swamp crayfish hepatopancreas reveals candidate genes in hif-1 and antioxidant pathways in response to hypoxia-reoxygenation. Mar. Biotechnol. 24 (1), 55–67. 10.1007/s10126-021-10086-9 34997878

[B235] Xu Z. Gao T. Xu Y. Li X. Li J. Lin H. (2021). A chromosome-level reference genome of red swamp crayfish *Procambarus clarkii* provides insights into the gene families regarding growth or development in crustaceans. Genomics 113 (5), 3274–3284. 10.1016/j.ygeno.2021.07.017 34303807

[B236] Yang Y. Fu Q. Wang X. Liu Y. Zeng Q. Li Y. (2018). Comparative transcriptome analysis of the swimbladder reveals expression signatures in response to low oxygen stress in channel catfish, *Ictalurus punctatus* . Physiol. Genomics 50 (8), 636–647. 10.1152/physiolgenomics.00125.2017 29799804

[B237] Yang Y. Zhou T. Liu Y. Tian C. Bao L. Wang W. (2022). Identification of an epigenetically marked locus within the sex determination region of channel catfish. Int. J. Mol. Sci. 23 (10), 5471. 10.3390/ijms23105471 35628283 PMC9171582

[B238] Yang Z. Wong S. M. Yue G. H. (2020). Characterization of GAB3 and its association with NNV resistance in the Asian seabass. Fish. Shellfish Immunol. 104, 18–24. 10.1016/j.fsi.2020.05.035 32473363

[B239] Yano A. Guyomard R. Nicol B. Jouanno E. Quillet E. Klopp C. (2012). An immune-related gene evolved into the master sex-determining gene in rainbow trout, *Oncorhynchus mykiss* . Curr. Biol. 22 (15), 1423–1428. 10.1016/j.cub.2012.05.045 22727696

[B240] Yano A. Nicol B. Jouanno E. Quillet E. Fostier A. Guyomard R. (2013). The sexually dimorphic on the Y-chromosome gene (sdY) is a conserved male-specific Y-chromosome sequence in many salmonids. Evol. Appl. 6 (3), 486–496. 10.1111/eva.12032 23745140 PMC3673476

[B241] Yuan Z. Liu S. Yao J. Zeng Q. Tan S. Liu Z. (2016). Expression of Bcl-2 genes in channel catfish after bacterial infection and hypoxia stress. Dev. Comp. Immunol. 65, 79–90. 10.1016/j.dci.2016.06.018 27353474

[B242] Zeng D. Guo X. (2022). Mantle transcriptome provides insights into biomineralization and growth regulation in the eastern oyster (*Crassostrea virginica*). Mar. Biotechnol. 24 (1), 82–96. 10.1007/s10126-021-10088-7 34989931

[B243] Zeng Y. Chen T. (2019). DNA methylation reprogramming during mammalian development. Genes (Basel). 10 (4), 257. 10.3390/genes10040257 PMC6523607 30934924

[B244] Zhang B. Zheng H. Huang B. Li W. Xiang Y. Peng X. (2016). Allelic reprogramming of the histone modification H3K4me3 in early mammalian development. Nature 537 (7621), 553–557. 10.1038/nature19361 27626382

[B245] Zhang G. Fang X. Guo X. Li L. Luo R. Xu F. (2012). The oyster genome reveals stress adaptation and complexity of shell formation. Nature 490 (7418), 49–54. 10.1038/nature11413 22992520

[B246] Zhang G. Li J. Zhang J. Liang X. Wang T. Yin S. (2020). A high-density SNP-based genetic map and several economic traits-related loci in *Pelteobagrus vachelli* . BMC Genomics 21 (1), 700. 10.1186/s12864-020-07115-7 33028208 PMC7542894

[B247] Zhang J. (2004). Evolution of DMY, a newly emergent male sex-determination gene of medaka fish. Genetics 166 (4), 1887–1895. 10.1534/genetics.166.4.1887 15126406 PMC1470821

[B248] Zhang T. Cooper S. Brockdorff N. (2015). The interplay of histone modifications - writers that read. EMBO Rep. 16 (11), 1467–1481. 10.15252/embr.201540945 26474904 PMC4641500

[B249] Zhang X. Li H. Qiu Q. Qi Y. Huang D. Zhang Y. (2014). 2, 4-Dichlorophenol induces global DNA hypermethylation through the increase of S-adenosylmethionine and the upregulation of DNMTs mRNA in the liver of goldfish *Carassius auratus* . Comp. Biochem. Physiol. C. Toxicol. Pharmacol. 160, 54–59. 10.1016/j.cbpc.2013.11.008 24316013

[B250] Zhang X. Yuan J. Sun Y. Li S. Gao Y. Yu Y. (2019). Penaeid shrimp genome provides insights into benthic adaptation and frequent molting. Nat. Commun. 10 (1), 356. 10.1038/s41467-018-08197-4 30664654 PMC6341167

[B251] Zhang Y. Liu Z. Li H. (2020). Genomic prediction of columnaris disease resistance in catfish. Mar. Biotechnol. 22 (1), 145–151. 10.1007/s10126-019-09941-7 31927643

[B252] Zhang Y. Shen W. Cao M. Li J. Zheng B. Lou Z. (2019). Dynamic alterations in methylation of global DNA and growth-related genes in large yellow croaker (*Larimichthys crocea*) in response to starvation stress. Comp. Biochem. Physiol. B Biochem. Mol. Biol. 227, 98–105. 10.1016/j.cbpb.2018.09.006 30315898

[B253] Zhong H. Xiao J. Chen W. Zhou Y. Tang Z. Guo Z. (2014). DNA methylation of pituitary growth hormone is involved in male growth superiority of Nile tilapia (*Oreochromis niloticus*). Comp. Biochem. Physiol. B Biochem. Mol. Biol. 171, 42–48. 10.1016/j.cbpb.2014.03.006 24704521

[B254] Zhong X. Wang X. Zhou T. Jin Y. Tan S. Jiang C. (2017). Genome-wide association study reveals multiple novel QTL associated with low oxygen tolerance in hybrid catfish. Mar. Biotechnol. 19 (4), 379–390. 10.1007/s10126-017-9757-5 28601969

[B255] Zhou M. Zhao Z. Zhao J. Wu M. Chen X. (2021). Gene expression profiling of DNA methyltransferase genes in *Siniperca chuatsi* based on transcriptome sequencing. J. Fish. Biol. 99 (5), 1755–1760. 10.1111/jfb.14862 34310718

[B256] Zhou T. Liu S. Geng X. Jin Y. Jiang C. Bao L. (2017). GWAS analysis of QTL for enteric septicemia of catfish and their involved genes suggest evolutionary conservation of a molecular mechanism of disease resistance. Mol. Genet. Genomics 292 (1), 231–242. 10.1007/s00438-016-1269-x 27826737

[B257] Zhu W. Xu X. Wang X. Liu J. (2019). Reprogramming histone modification patterns to coordinate gene expression in early zebrafish embryos. BMC Genomics 20 (1), 248. 10.1186/s12864-019-5611-7 30922236 PMC6437866

